# SPRINT Through Tasks: A Novel Curriculum for Improving Resident Task Management in the Emergency Department

**DOI:** 10.15766/mep_2374-8265.10956

**Published:** 2020-08-25

**Authors:** Brett R. Todd, Stephanie Traylor, Leah Heron, Danielle Turner-Lawrence

**Affiliations:** 1 Assistant Professor, Department of Emergency Medicine, Oakland University William Beaumont School of Medicine; 2 Physician, Department of Emergency Medicine, Mount Carmel St. Ann's Hospital, Westerville, Ohio; 3 Clinical Assistant Professor, Department of Emergency Medicine, Michigan State University College of Human Medicine; 4 Associate Professor, Department of Emergency Medicine, Oakland University William Beaumont School of Medicine

**Keywords:** Task Switching, Task Management, Multitasking, Interruptions, Efficiency, Decision Fatigue, Clinical Reasoning/Diagnostic Reasoning, Quality Improvement/Patient Safety, Emergency Medicine, Games, Multimedia

## Abstract

**Introduction:**

The emergency department (ED) presents a challenging task-management environment to emergency medicine (EM) trainees. However, equipping residents with a tool to improve task switching (generically known as multitasking) could have positive impacts on patient care and physician emotional state. We designed a task-management tool and educational curriculum with the goal of improving emergency medicine resident task-switching ability.

**Methods:**

The task-management tool uses the acronym SPRINT: (1) stabilize critical patients, (2) perform procedures, (3) rack (see new patients in the chart rack), (4) in or out (reassess and disposition), (5) type it up (chart completion). These tasks and their order were decided on by two seasoned clinicians based on their years of experience in the ED. The SPRINT tool was taught to EM residents through a 1-hour curriculum consisting of an introductory video, a classroom-based workshop with multimedia didactics, and team learning with a card game simulating the use of the SPRINT tool on a shift. Residents were surveyed to evaluate their task-management confidence and perceived effectiveness of the curriculum.

**Results:**

A total of 34 EM residents participated in this training on the SPRINT tool. There was an improvement in resident confidence in task management, and residents reporting having a strategy for task prioritization 8 weeks after the workshop.

**Discussion:**

The SPRINT curriculum provides EM residents with a tool to manage the complex task-management environment of the ED. Further research in task-management education should focus on patient-oriented outcomes among physicians who have received this training.

## Educational Objectives

By the end of this activity, residents working in the emergency department (ED) will be able to:

1. Describe multitasking/task switching, and its impacts on clinical performance.
2. Describe the ways in which the ED environment presents the clinician with unique decision-making challenges due to the necessity of multitasking and frequent interruptions.
3. Apply a task-management system while working in the ED designed to improve their workflow and help manage issues relating to multitasking.

## Introduction

Emergency physicians (EPs) work in a challenging decision-making environment, which requires addressing multiple tasks simultaneously and in a compressed span of time; a problem widely known in the emergency medicine (EM) community as multitasking. We will use the terms *task switching* and *multitasking* interchangeably. Specifically, the EP must quickly evaluate multiple patients, manage a wide range of complex medical conditions and acuities, and interact with many individuals on any given shift.^1^ This presents the EP with an astronomical number of possible orders in which to complete their tasks. For example, during a busy shift in the emergency department (ED), the EP may be faced with eight patients needing disposition, four patients who need to be seen, and two procedures yet to be performed. These 14 tasks can be performed in (14)! or 87,178,291,200 different possible combinations.

The strain of multitasking on EP decision-making is made worse by the adverse task-management environment of the ED, characterized by frequent interruptions,^2^ ED overcrowding,^3^ and excessive noise.^4^ The EP is therefore presented with an exceptionally large number of tasks to accomplish at any given time (a problem known as decision fatigue) and must handle these tasks in a setting poorly conducive to effective decision making. This combination of a large task load and frequent interruptions has been demonstrated to have negative effects on patient care, physician judgement, and physician emotional state.^4,5^ Most concerning, the detriment to quality decision making can contribute to medical error.^6^

EM trainees may be particularly stressed by the adverse task load they face while on shift due to their lack of medical knowledge, limited clinical experience, and the demands of training including long work hours and educational requirements. Multitasking and frequent interruptions have been associated with EM resident fatigue, stress, and burnout.^7^ Educating residents on the management of multitasking by training in task switching is an obligation of EM residency programs, and is a specific skill required for resident evaluation as described in the ACGME EM Milestone Project.^8^

Unfortunately, little is known about how to best educate residents on multitasking skills. Prior published attempts at educating residents on managing multitasking have used simulation with limited success.^9^ Various cognitive strategies have been proposed to mitigate the adverse effects of interruptions^10^ and limit physician error,^11^ but, to our knowledge, have not been evaluated as a way to improve multitasking ability in residents in the ED. In addition, prior published educational curricula have simulated task saturation, however, to our knowledge, none have utilized specific strategies for managing simultaneous tasks.^12,13^ We developed a cognitive tool, referred by the acronym SPRINT, to assist EM residents with the goal of improving resident's efficiency and confidence with managing tasks on shift. The SPRINT tool guided residents through their task list in a systematic way and ensured that the highest-priority tasks were managed first. It was adaptable to changes in patient status and accommodated new tasks when they arose. We then designed an interactive curriculum for educating EM residents on the use of the SPRINT tool to improve their multitasking skills. The SPRINT tool was taught to residents at our EM residency using the curriculum during a 1-hour period at our weekly educational conference.

## Methods

We developed a simple clinical decision tool called SPRINT with the purpose of guiding residents through the vast number of tasks they must complete while on shift in the ED. The SPRINT tool instructs residents to manage their tasks in the following order: (1) stabilize critical patients, (2) perform procedures, (3) rack (see new patients in the chart rack), (4) in or out (reassess and disposition), (5) type it up (chart completion). When new tasks arose, the resident was instructed to place that task in the proper order according to the SPRINT tool. These steps were based on the workflow experience of the study authors, who noticed EM residents struggling with the many tasks requiring completion in the ED. The authors have over 30 years of combined clinical experience across multiple EDs throughout the United States and have found that ordering tasks in this manner has increased their efficiency while lowering their stress due to multitasking. This task ordering was translated into the SPRINT mnemonic. The tool was designed to address the most clinically important tasks first (critically ill patients), followed by the most time-consuming task (procedures), followed by keeping up with departmental flow (seeing new patients and dispositioning active patients), and finally completing charting when the opportunity arose.

The Oakland University William Beaumont Institutional Review Board approved the teaching and assessment of the SPRINT tool through a novel educational curriculum to EM residents at our 3-year EM residency program, located in a 130,000-visit, suburban, level-one trauma ED. The SPRINT curriculum was taught over a 1-hour workshop during our weekly resident educational conference. A multimodal, interactive educational curriculum was used. The curriculum consisted of a brief didactic lecture, an educational video using simulated clinical experiences, an interactive small-group card game demonstrating the proper use of the SPRINT tool, and a 5-minute summary with a question-and-answer period.

### Structure of the Educational Workshop

#### Time 0 to 15 minutes

Residents first received a 15-minute lecture (Appendix A) introducing them to the concept of multitasking and its negative consequences on physician performance including decision fatigue, cognitive overload, stress, and errors. The lecture educated them on the consequences of multitasking and the particular ways in which the ED adversely affects decision making. The SPRINT tool was introduced briefly in the lecture.

#### Time 15 to 20 minutes

A 5-minute video (Appendix B) was shown, which explained the SPRINT method for task management. The video presented a short skit in which a resident gets overwhelmed by her ED tasks, and then re-runs the skit with her successfully completing her tasks using the SPRINT tool. This provided the learners with a visual understanding of how the SPRINT tool is used and how it can benefit them.

#### Time 20 to 25 minutes

We then reinforced the lecture by reviewing the SPRINT steps and introduced a card game to be played to simulate task management on shift using the SPRINT methodology.

#### Time 25 to 55 minutes

The residents spent the next 30 minutes playing the card game (Appendix C). Learners were split up into groups of two to three residents, and each group was given a deck of cards. Each playing card had a brief clinical vignette which described a task to be completed. The groups shuffled the card deck and dealt five cards representing five tasks to be completed and were instructed to place the cards in the proper order using the SPRINT tool. The first card in order (representing the highest priority task) was then removed, which represented a completed task. A card was then drawn from the top of the deck, and then had to be placed in the correct order with the remaining four cards. This process was repeated until all of the cards in the deck were used, representing all tasks completed for the shift. Three faculty proctors were present to assist the residents with the card game and to answer any questions about the proper ordering of tasks. It was emphasized that the SPRINT tool is a guideline that applies in almost any clinical scenario, however clinical judgement can overrule the tool when prudent. An example of a reasonable break from the SPRINT format would be to take 2 minutes to discharge a patient with an ankle sprain before spending 45 minutes performing a lumbar puncture.

#### Time 55 to 60 minutes

The end of the hour was used to summarize the main learning points, review the SPRINT tool, and the residents' experience trying the tool while utilizing the card game. Additionally, faculty answered any remaining questions from the learners about the SPRINT tool and its application clinically. Each resident was also given a laminated badge card (Appendix D) containing the instructions for using the SPRINT tool as a readily available reference while working clinically in the ED.

### Assessment

The effectiveness of the SPRINT curriculum was assessed using anonymous pre- and postworkshop surveys (Appendices E and F). The surveys utilized a 5-point Likert scale (1 = *strongly disagree, 3* = *neutral,* 5 = *strongly agree)* to assess the residents' confidence in task management and opinions about the SPRINT tool and educational curriculum. The preworkshop survey had six questions, and the postworkshop survey included three additional questions assessing the enjoyment of the workshop and resident application of the SPRINT tool. The postworkshop electronic survey was sent to participants 8 weeks after the workshop, using Survey Monkey. Mean Likert scores for pre- and postworkshop surveys were calculated by PGY level and compared using a student's *T* test. Pre- and postworkshop survey paired questions (1–6) were dichotomized to positive (Likert items 4 and 5) and neutral/negative (Likert items 1, 2, and 3) and then compared by chi-squared test. Significance was considered when the *p* value was <.05.

## Results

A total of 23 EM residents attended the SPRINT workshop, and an additional 11 EM residents viewed the video online but did not attend the workshop. Of participants, 73% found the educational activity enjoyable. Immediately after the educational activity, 84% of residents responded that they planned on using the SPRINT tool while on shift. On the 8-week postworkshop follow-up survey, 63% of respondents replied that they had in fact used the tool while on shift. Of residents, 54% agreed or strongly agreed with the statement, “I feel more confident after attending the workshop or watching the video on multitasking” 8 weeks after the workshop.

Likert scores from the pre- and postworkshop surveys showed non-significant trends towards improvement in satisfaction with the ability to manage tasks efficiently, feeling overwhelmed by the number of tasks, and the belief that a decision tool is effective with task management (Figure). There were no significant differences in Likert survey responses by PGY level (Table). However, on analysis of responses dichotomized to negative/neutral and positive, there was a significant improvement in residents reporting that they had a strategy for task management (chi-square = 5.07, *p* < .024), and this result was mostly attributable to PGY 1 improvement (chi-square = 6.17, *p* < .013).

**Figure.**
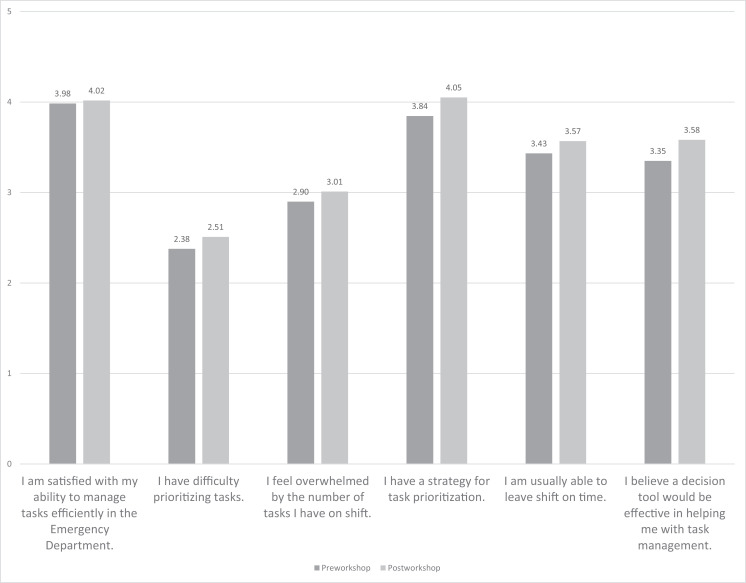
Preworkshop vs. postworkshop survey mean Likert-scale data—all PGY levels.

**Table.**
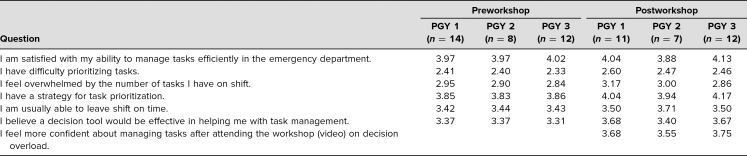
Likert Scale Mean per PGY Level

## Discussion

Our educational curriculum was the first to attempt to improve resident task switching ability in the ED. EPs are confronted with an extremely high number of tasks to complete while on shift, with one observational study recording 101 discrete tasks performed per hour by EPs.^14^ This vast number of tasks requires the development of advanced multitasking skills in EM residents. In fact, the ACGME EM Milestones Project includes a multitasking competency requirement for graduating EM residents.^8^ Prior studies have demonstrated that EM resident productivity increases year by year,^15^ and even on a monthly basis.^16^ However, this improvement appears to result from clinical experience in the ED, as there are no educational curricula published addressing this skill. Successful management of multitasking requires ordering tasks by urgency as well as by improving patient throughput in the ED. The SPRINT tool provided a roadmap through this enormous number of tasks to be accomplished by an EP. Therefore, it functioned as a cognitive debiasing tool, which may help with the reduction of error.^17^ This educational curriculum explained the SPRINT tool to residents, demonstrated the tool's use in a brief video, and gave the residents an opportunity to practice using the tool with a team-based card game.

Our results showed that an educational curriculum focusing on the development of multitasking skills in EM residency had the potential to improve resident task-management ability. Residents were more likely to report having a strategy to manage tasks in the ED after receiving training in the SPRINT decision tool, and this effect was most notable in PGY 1 residents who may have more difficulty managing the chaotic environment of the ED due to their lack of clinical experience. Additionally, residents felt more confidence with task management after undergoing training using the SPRINT tool. These results were encouraging given the known negative consequences of multitasking on physician emotional state and patient care.

Our study was limited in that it was performed at a single residency site and at one hospital ED, which may limit the generalizability of our results. Additionally, the curriculum was presented only one time to our residents during the educational year. The small sample size of participants may have limited the ability to detect meaningful improvement in task management. The curriculum was provided by video to the 32% of our residents who were unable to attend the workshop, which may have limited their comprehension and retention of material. Furthermore, the gaming component of the workshop may not be a learning style that is effective for all residents. Most residents did, however, enjoy the game component. The card game we implemented used images on the playing cards which were specific to our hospital training site. These images were removed for publication so that the card game could be more generalizable, but this aspect may contribute to the enjoyment of the game: We encourage users of the game to insert their own images to make it more relevant and engaging for their trainees. Repeated brief educational sessions covering multitasking and managing task switching with the SPRINT tool may result in a greater effect on task-management abilities. Lastly, the study survey did not address the effects of the SPRINT tool on physician emotional state, patient care outcomes, or efficiency outcomes such as waiting times and relative value units (RVUs).

Future efforts in task-management education may focus on early learners in EM, who benefited the most from this training in our study. The clinical impact of the SPRINT tool and other task management curricula need to be evaluated in future studies, possibly by looking at effects on resident productivity as measured by patients seen per hour, or RVUs.

## Appendices

Task Management in the ED.pptxSPRINT Video.mp4SPRINT Card Game.pptxSPRINT Badge Card.pdfSPRINT Preworkshop Survey.docxSPRINT Postworkshop Survey.docx
All appendices are peer reviewed as integral parts of the Original Publication.
