# Supplementary material for: SPRINT Through Tasks: A Novel Curriculum for Improving Resident Task Management in the Emergency Department
Source: MedEdPORTAL. 2020 Aug 25;16:10956. doi: 10.15766/mep_2374-8265.10956 (PMC7449580; doi:10.15766/mep_2374-8265.10956)
Supplement: Supplementary file 1 — Task Management in the ED.pptxSPRINT Video.mp4SPRINT Card Game.pptxSPRINT Badge Card.pdfSPRINT Preworkshop Survey.docxSPRINT Postworkshop Survey.docx [file mep_2374-8265.10956-s001.zip › A. Task Management in the ED.pptx]

## Slide 1
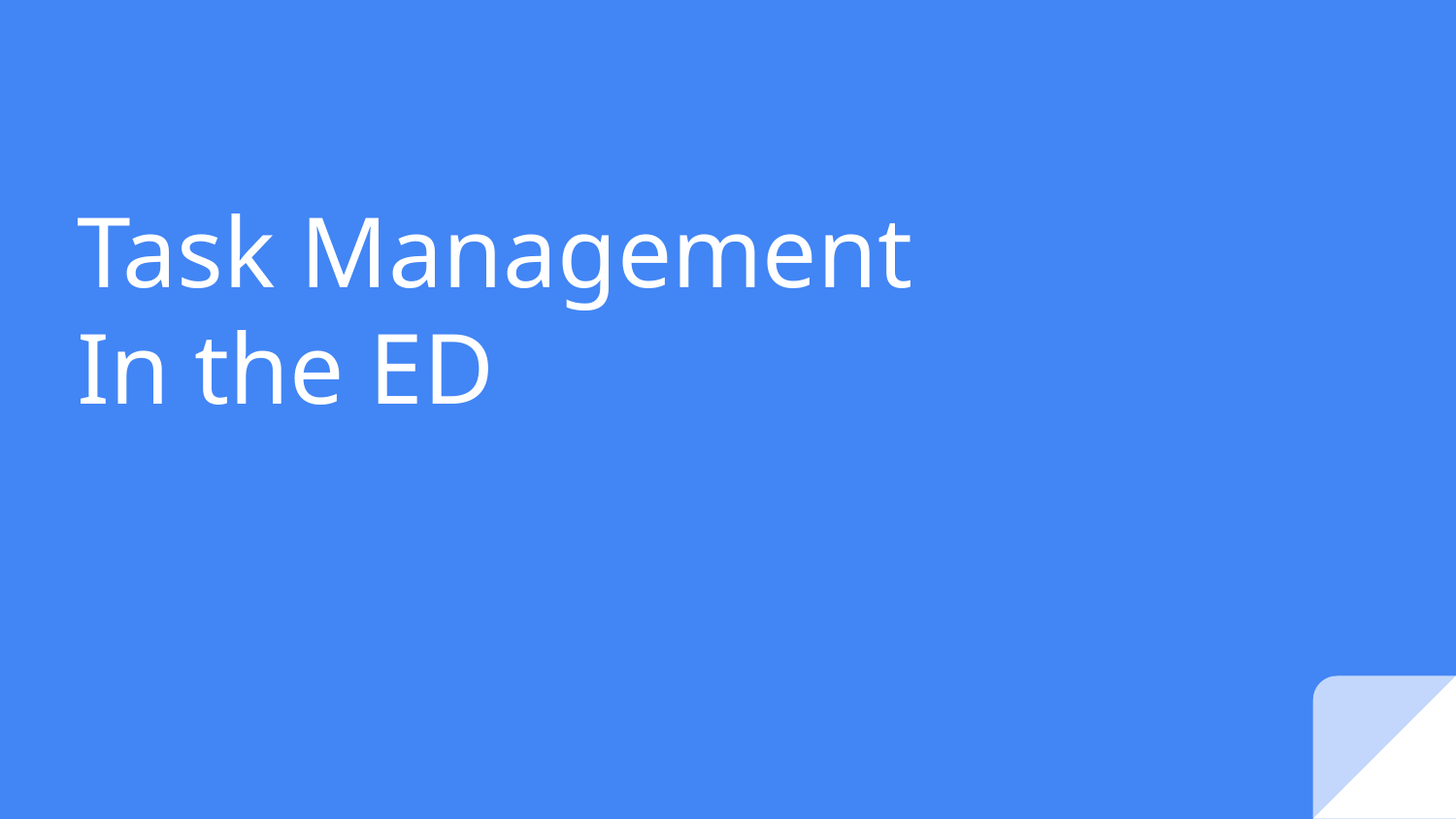

# Task Management
In the ED

## Slide 2
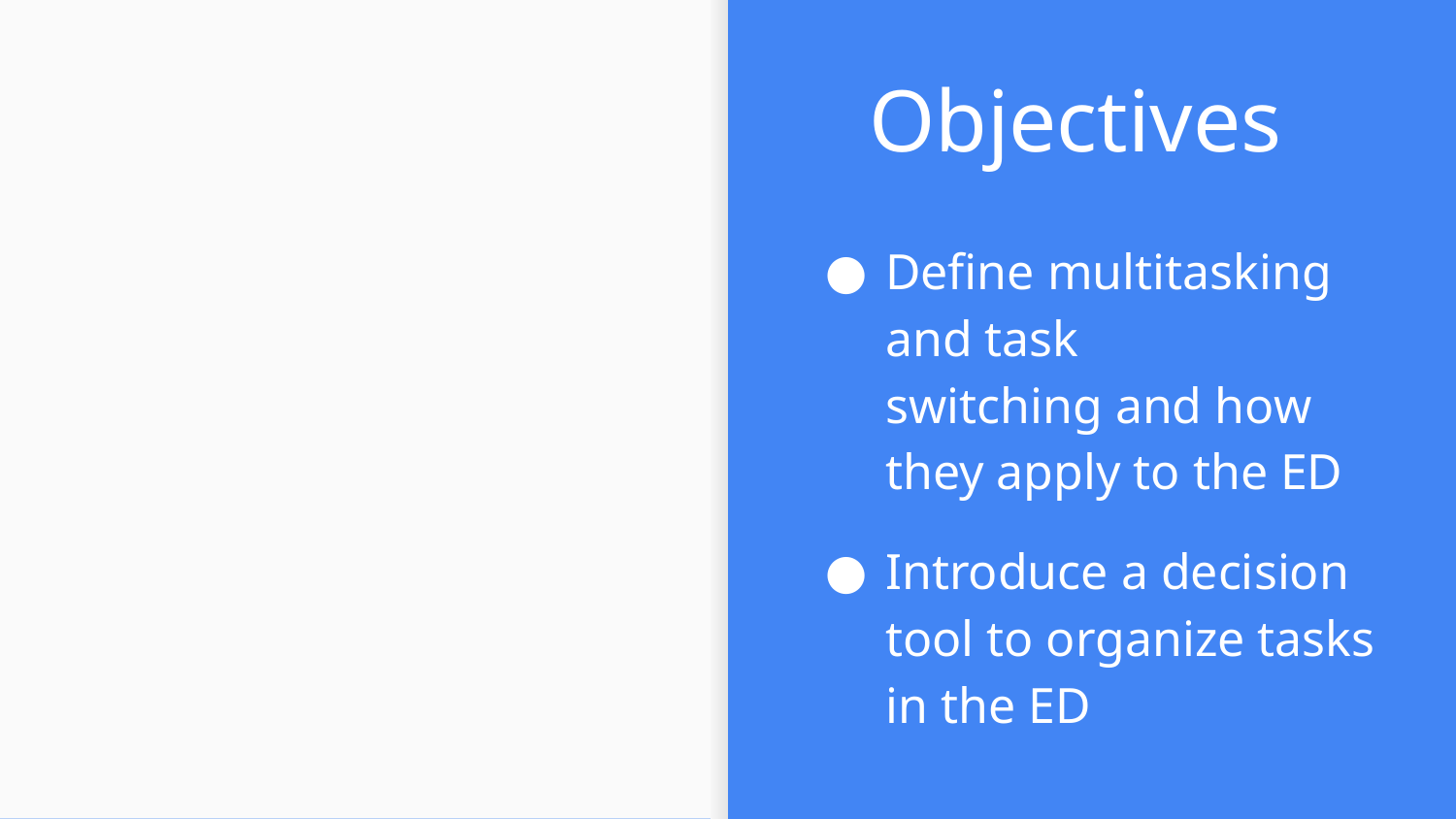

# Objectives
Define multitasking and task switching and how they apply to the ED
Introduce a decision tool to organize tasks in the ED

## Slide 3
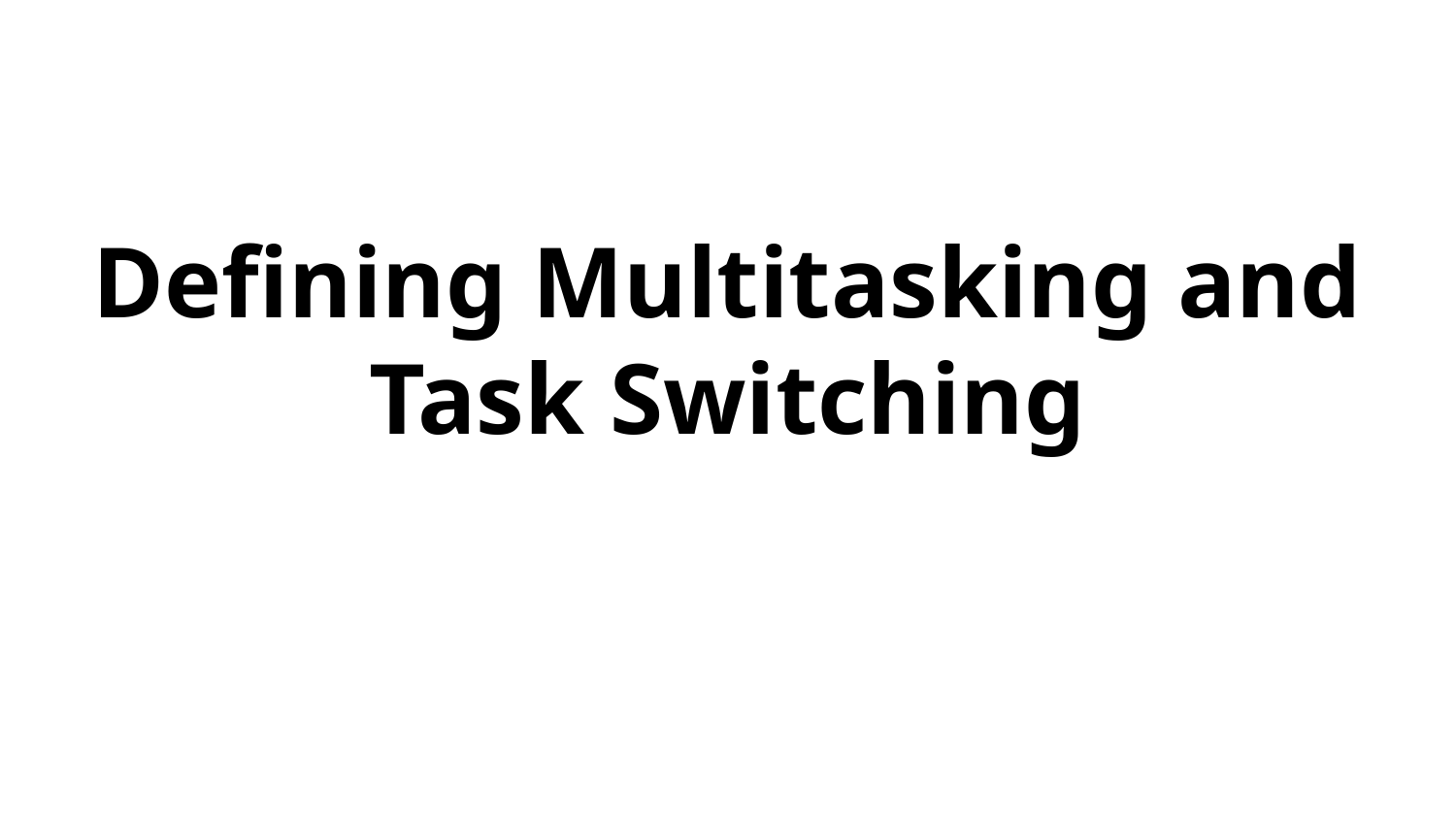

# Defining Multitasking and Task Switching

## Slide 4
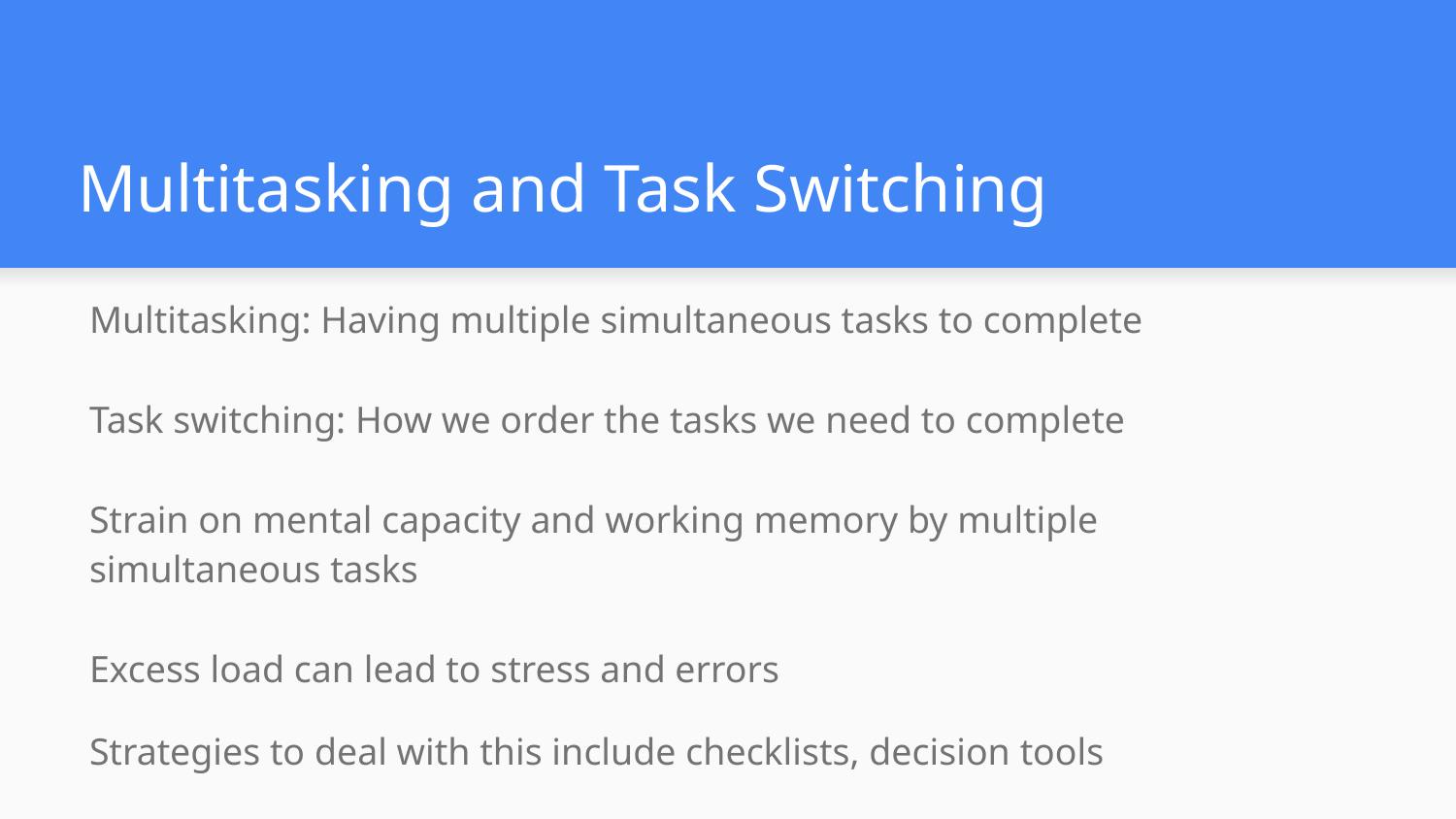

# Multitasking and Task Switching
Multitasking: Having multiple simultaneous tasks to complete
Task switching: How we order the tasks we need to complete
Strain on mental capacity and working memory by multiple simultaneous tasks
Excess load can lead to stress and errors
Strategies to deal with this include checklists, decision tools

## Slide 5
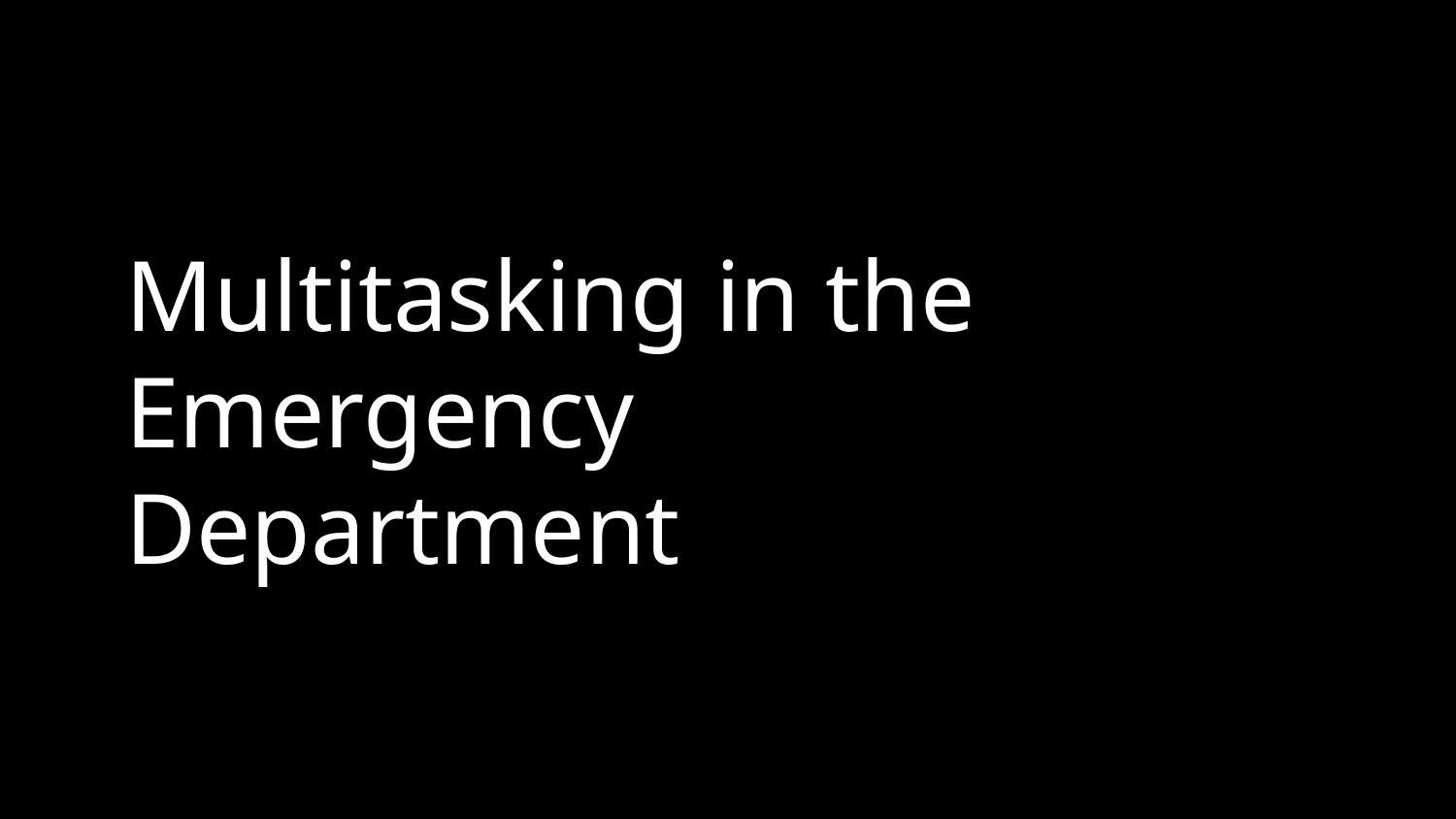

# Multitasking in the Emergency Department

## Slide 6
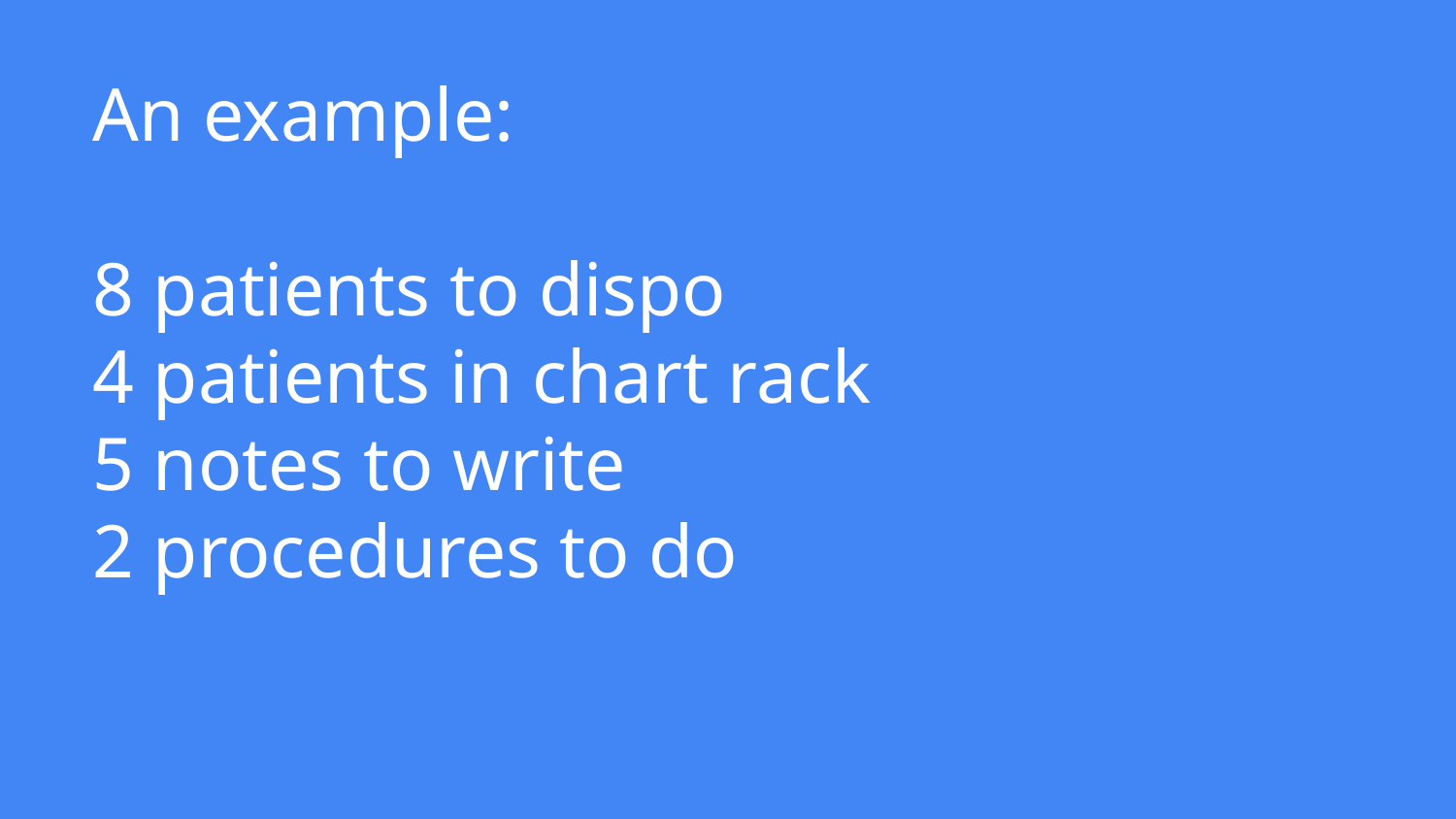

# An example:
8 patients to dispo
4 patients in chart rack
5 notes to write
2 procedures to do

## Slide 7
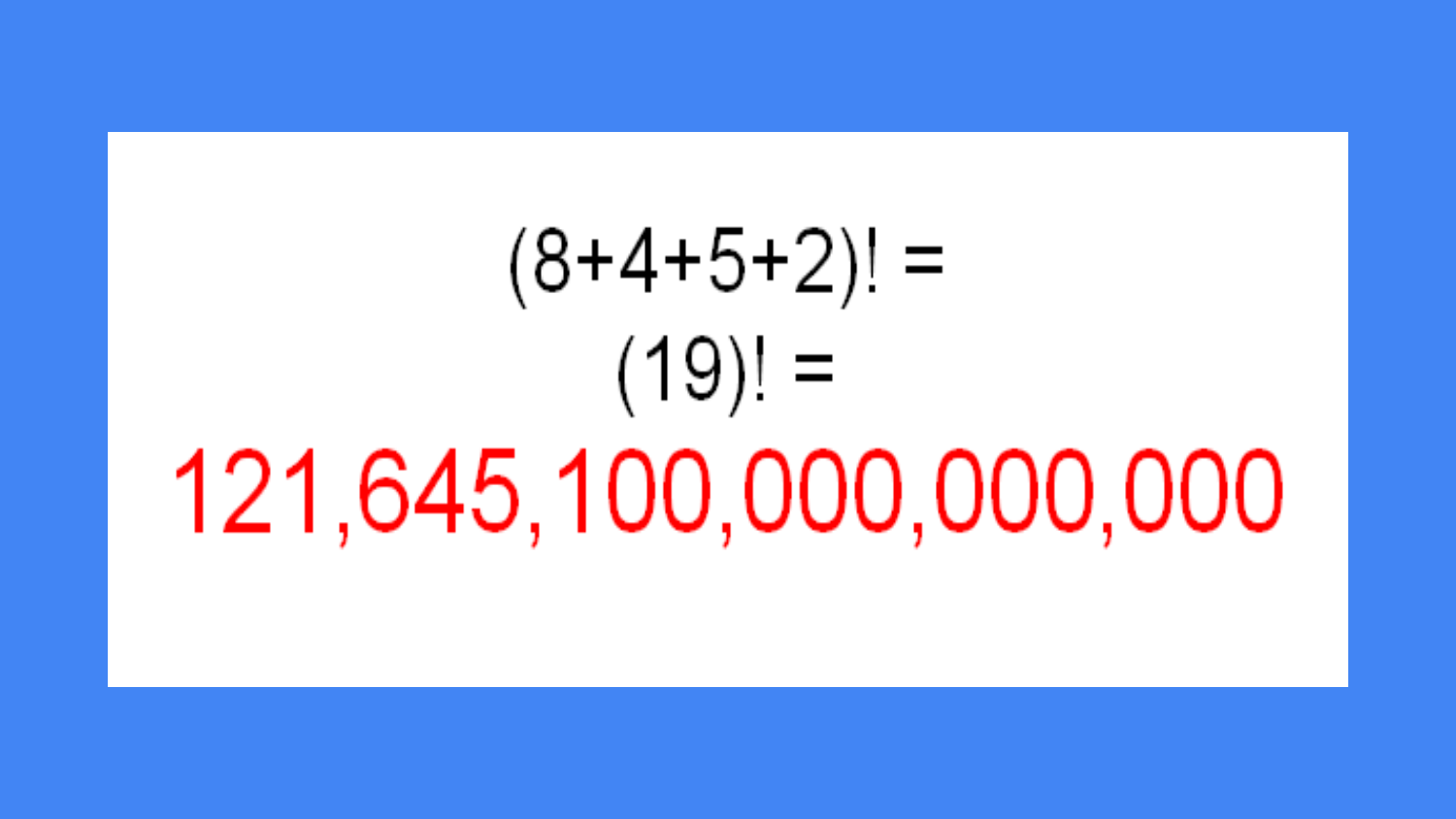

## Slide 8
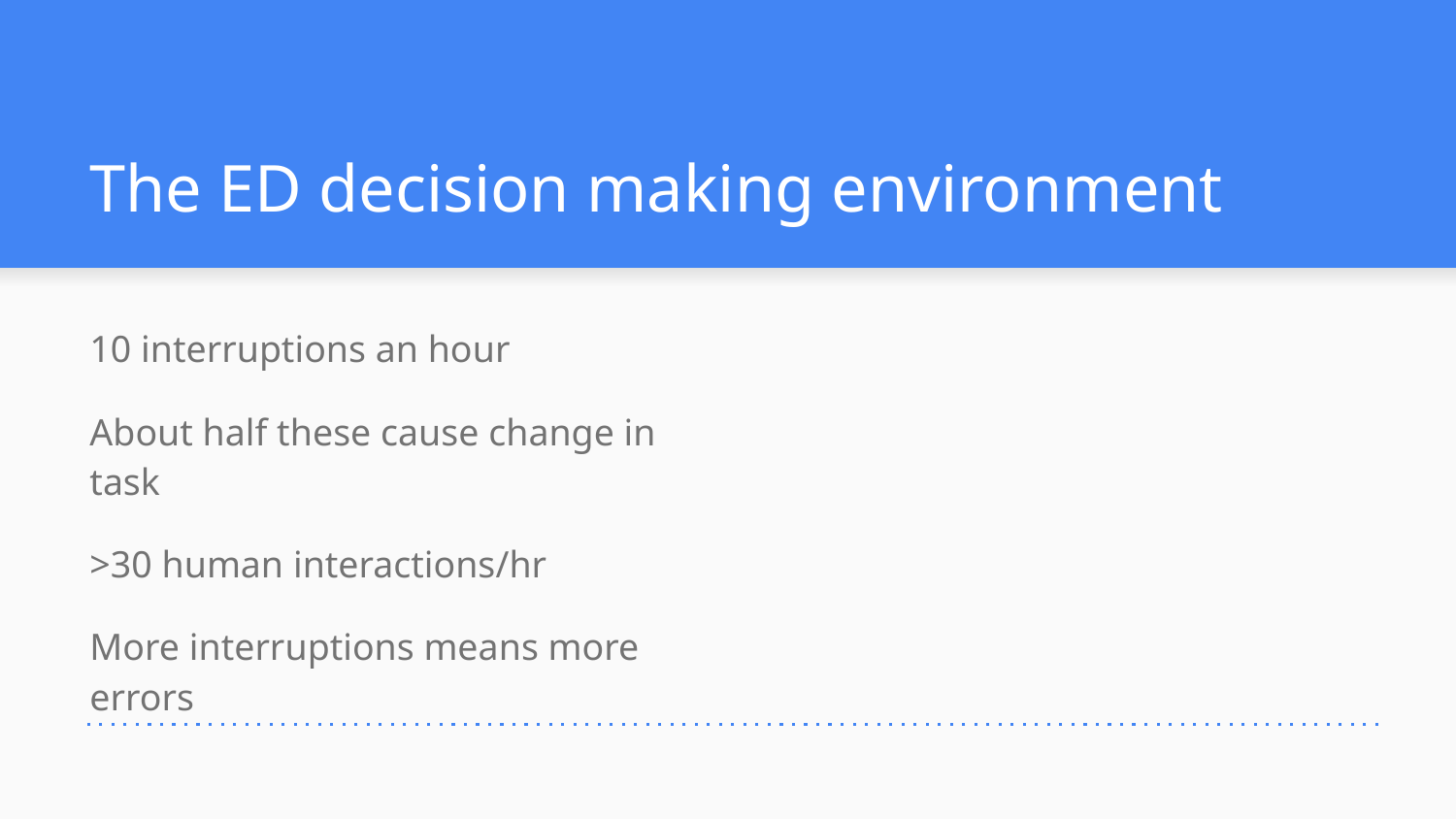

# The ED decision making environment
10 interruptions an hour
About half these cause change in task
>30 human interactions/hr
More interruptions means more errors

## Slide 9
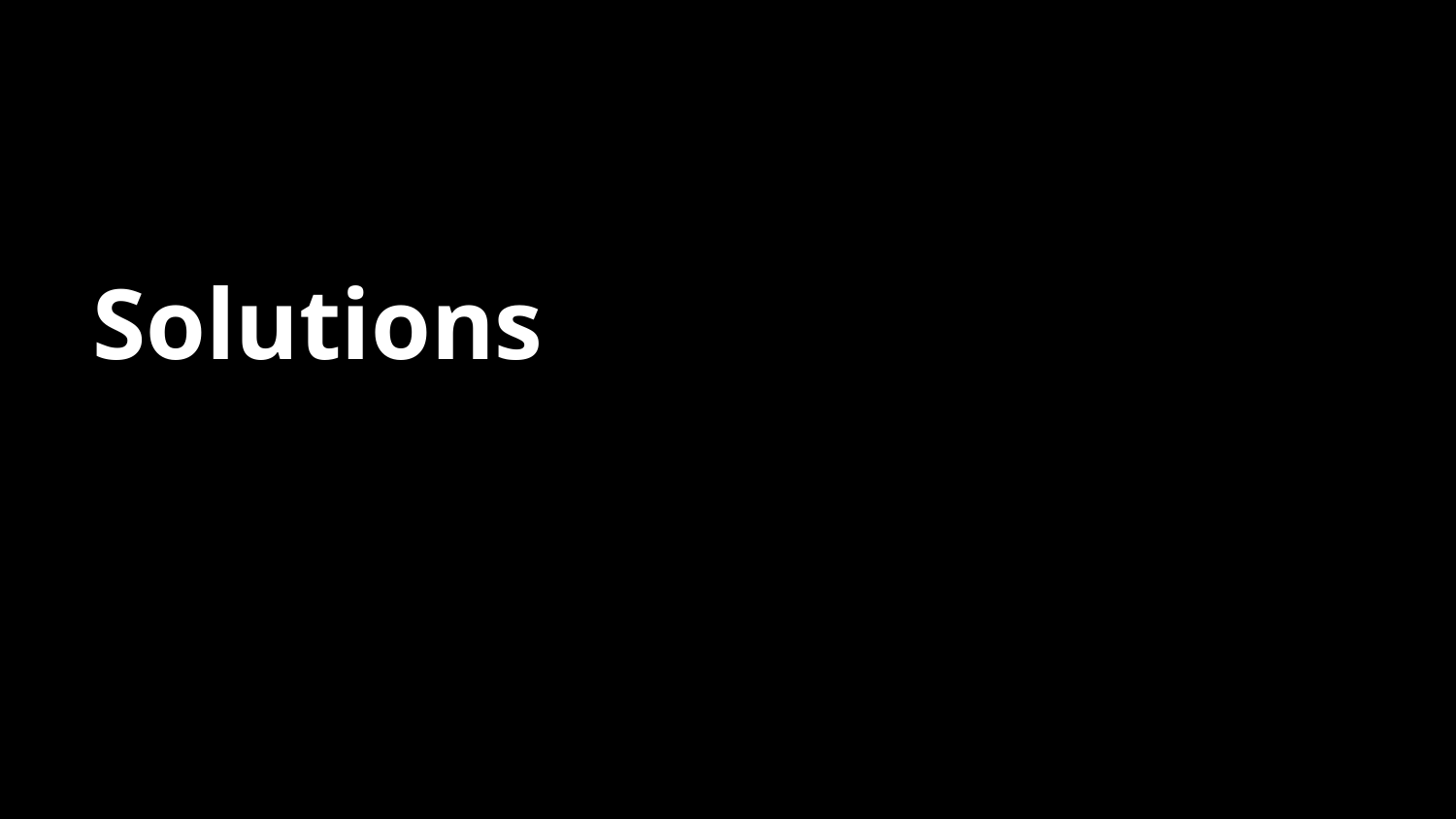

# Solutions

## Slide 10
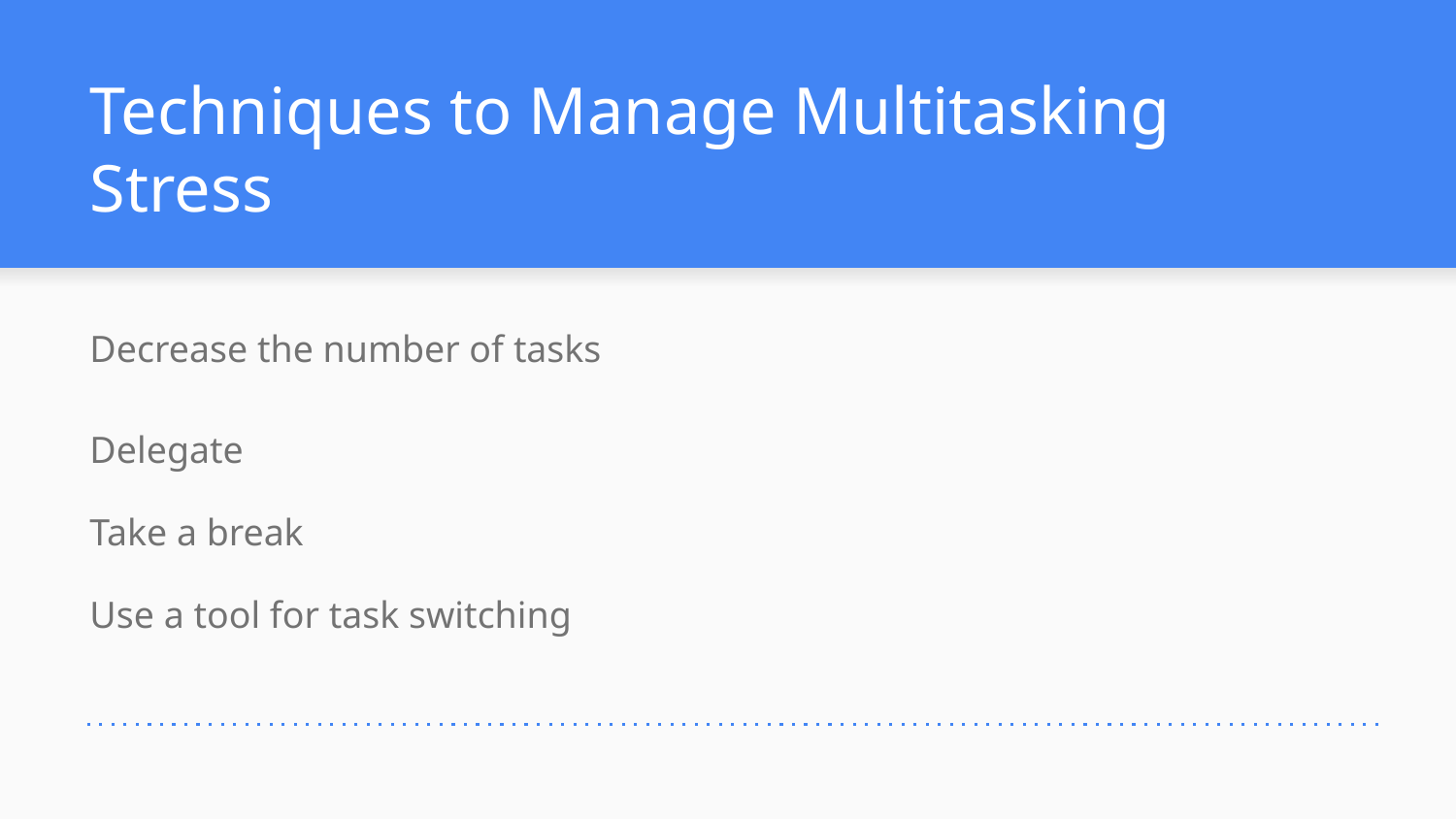

# Techniques to Manage Multitasking Stress
Decrease the number of tasks
Delegate
Take a break
Use a tool for task switching

## Slide 11
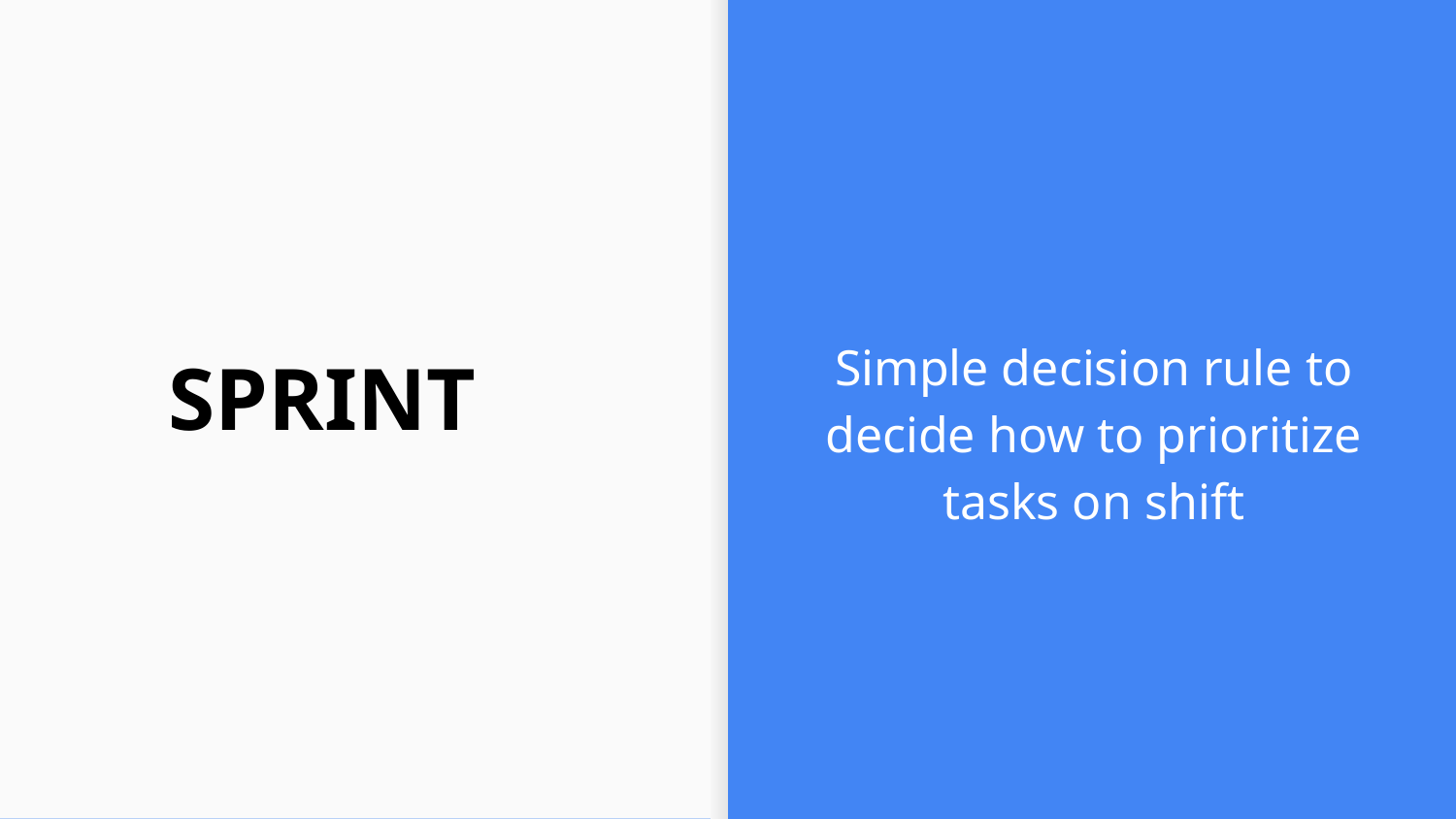

Simple decision rule to decide how to prioritize tasks on shift
# SPRINT

## Slide 12
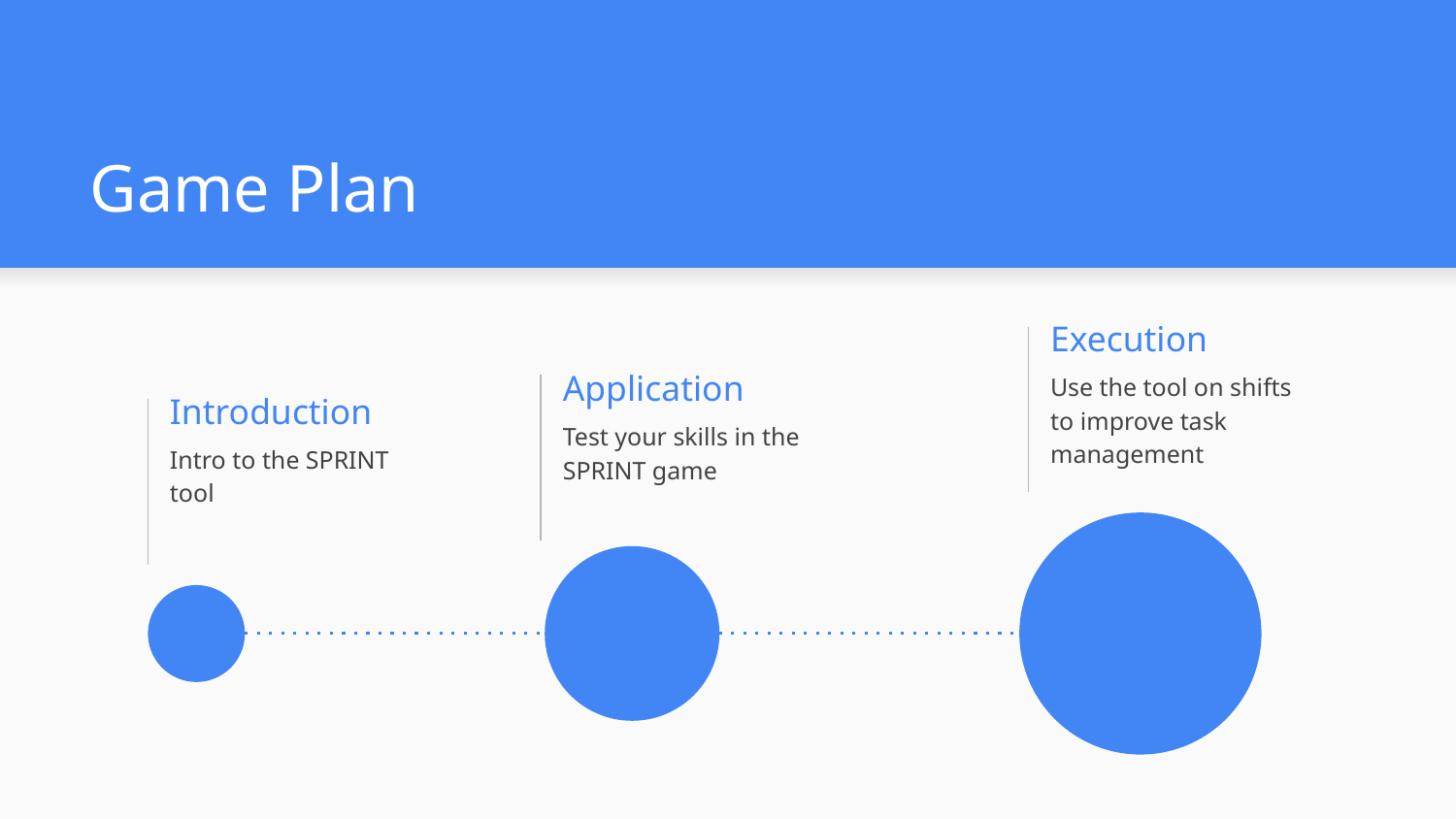

# Game Plan
Execution
Use the tool on shifts to improve task management
Application
Introduction
Test your skills in the SPRINT game
Intro to the SPRINT tool

## Slide 13
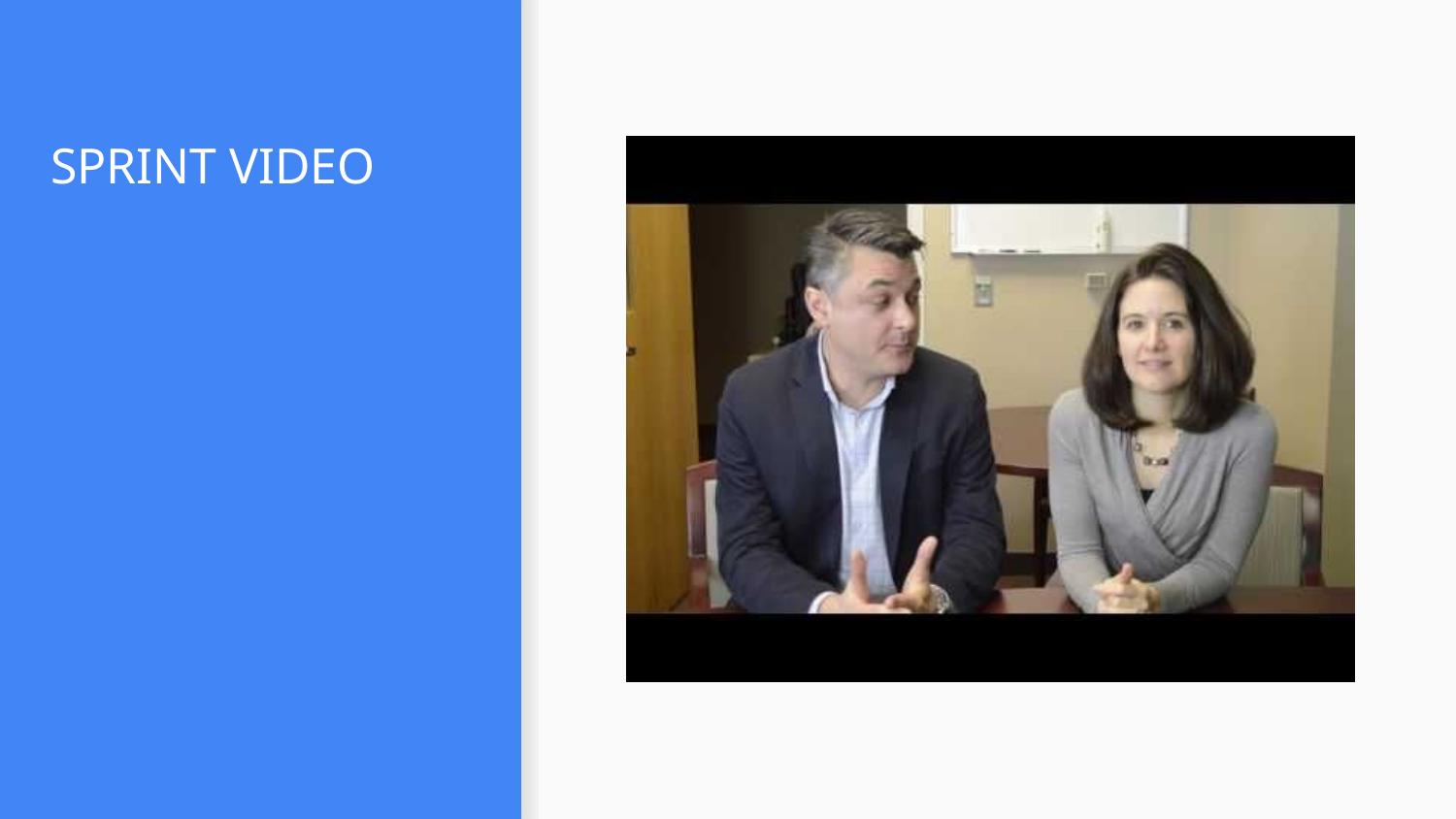

# SPRINT VIDEO

## Slide 14
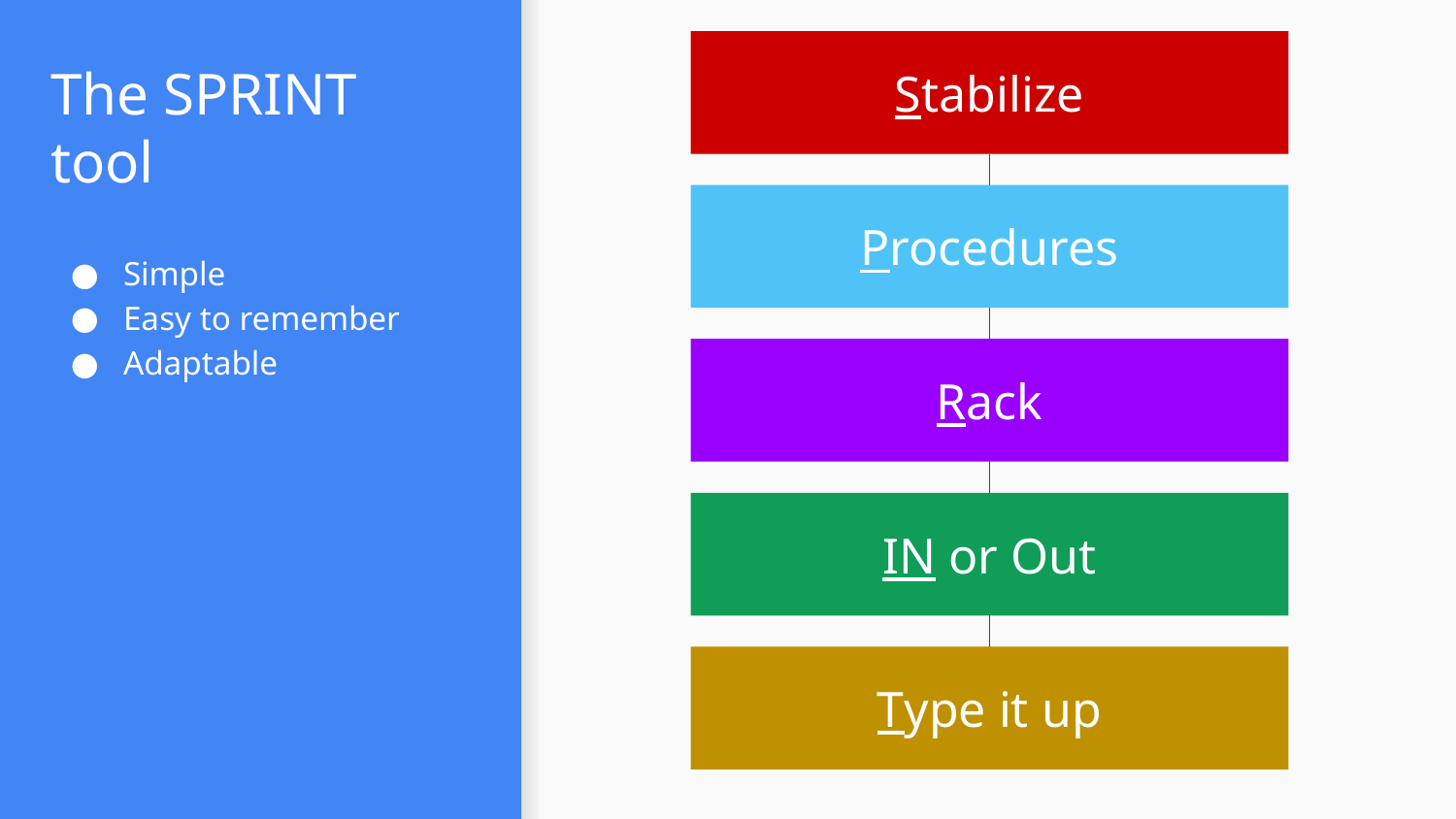

Stabilize
# The SPRINT tool
Procedures
Simple
Easy to remember
Adaptable
Rack
IN or Out
Type it up

## Slide 15
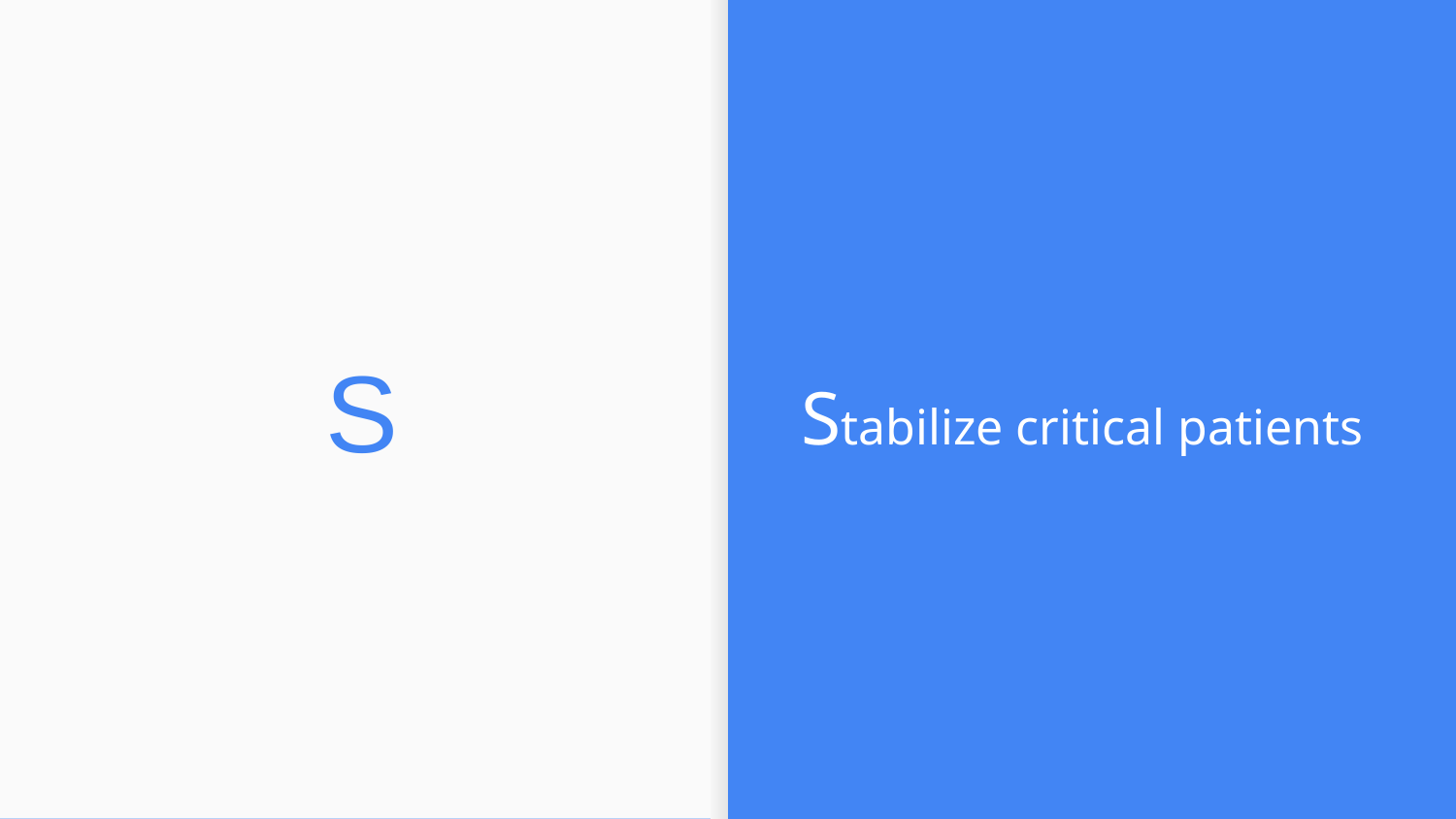

Stabilize critical patients
S

## Slide 16
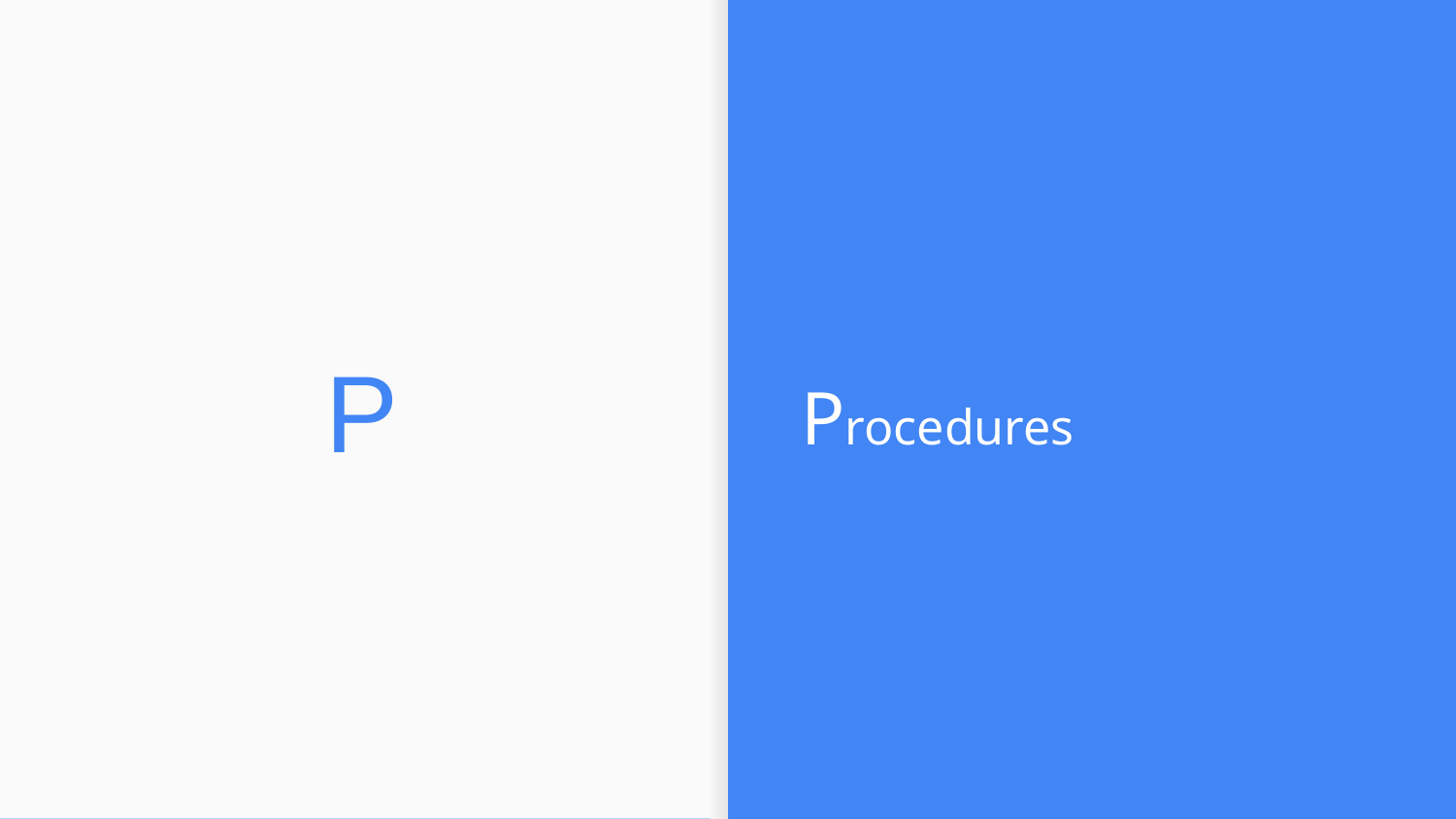

Procedures
P

## Slide 17
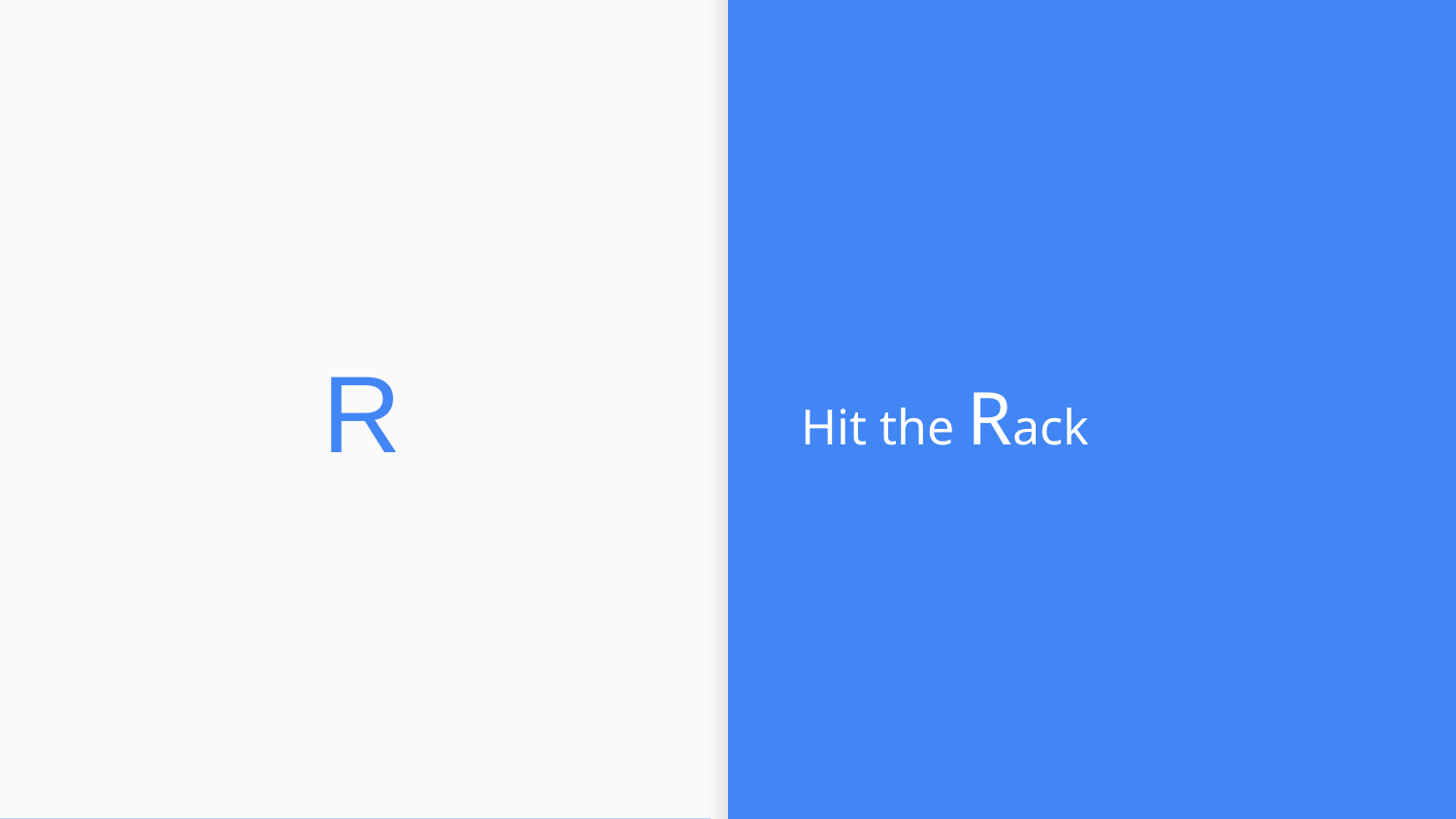

Hit the Rack
R

## Slide 18
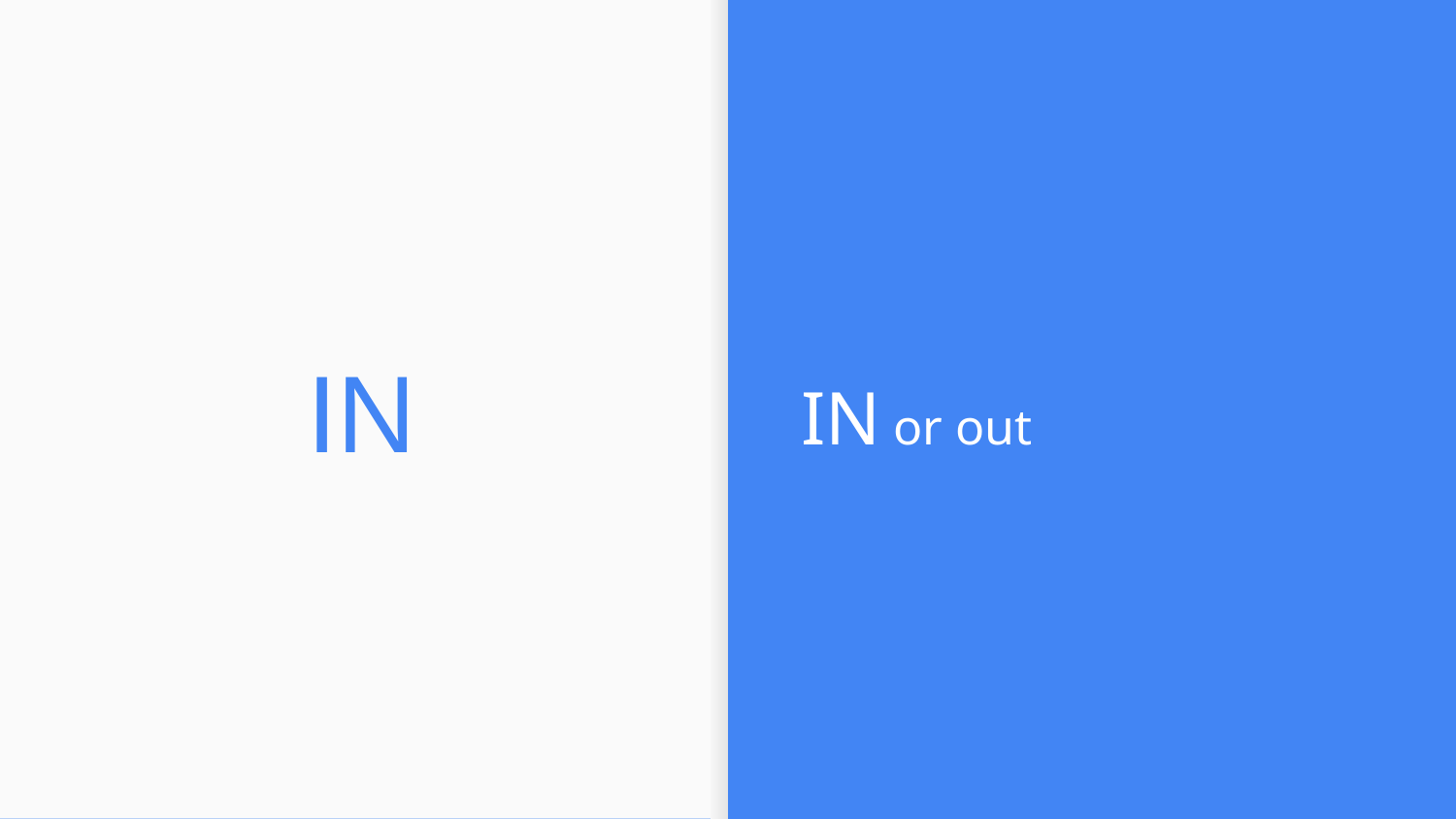

IN or out
IN

## Slide 19
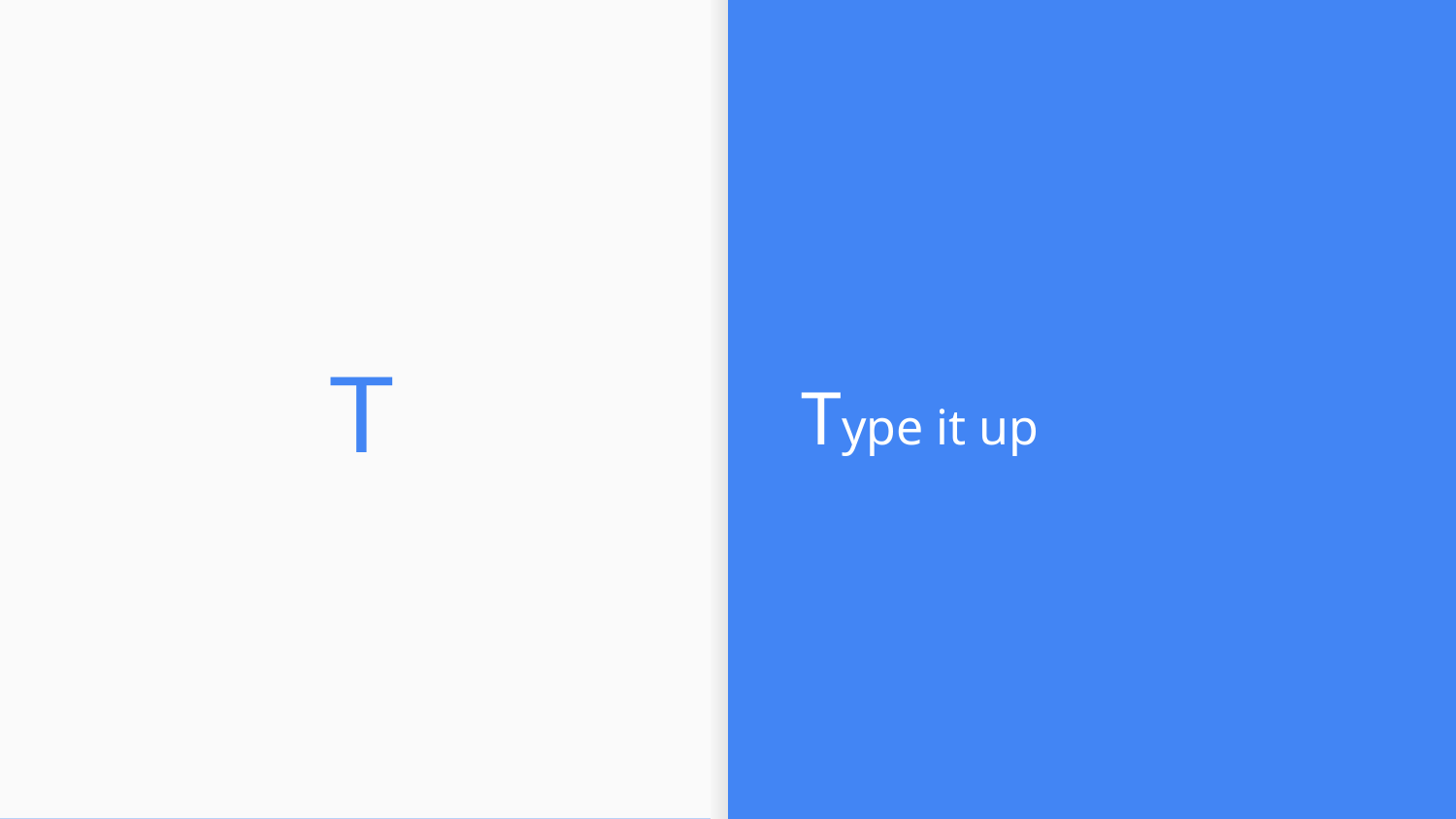

Type it up
T

## Slide 20
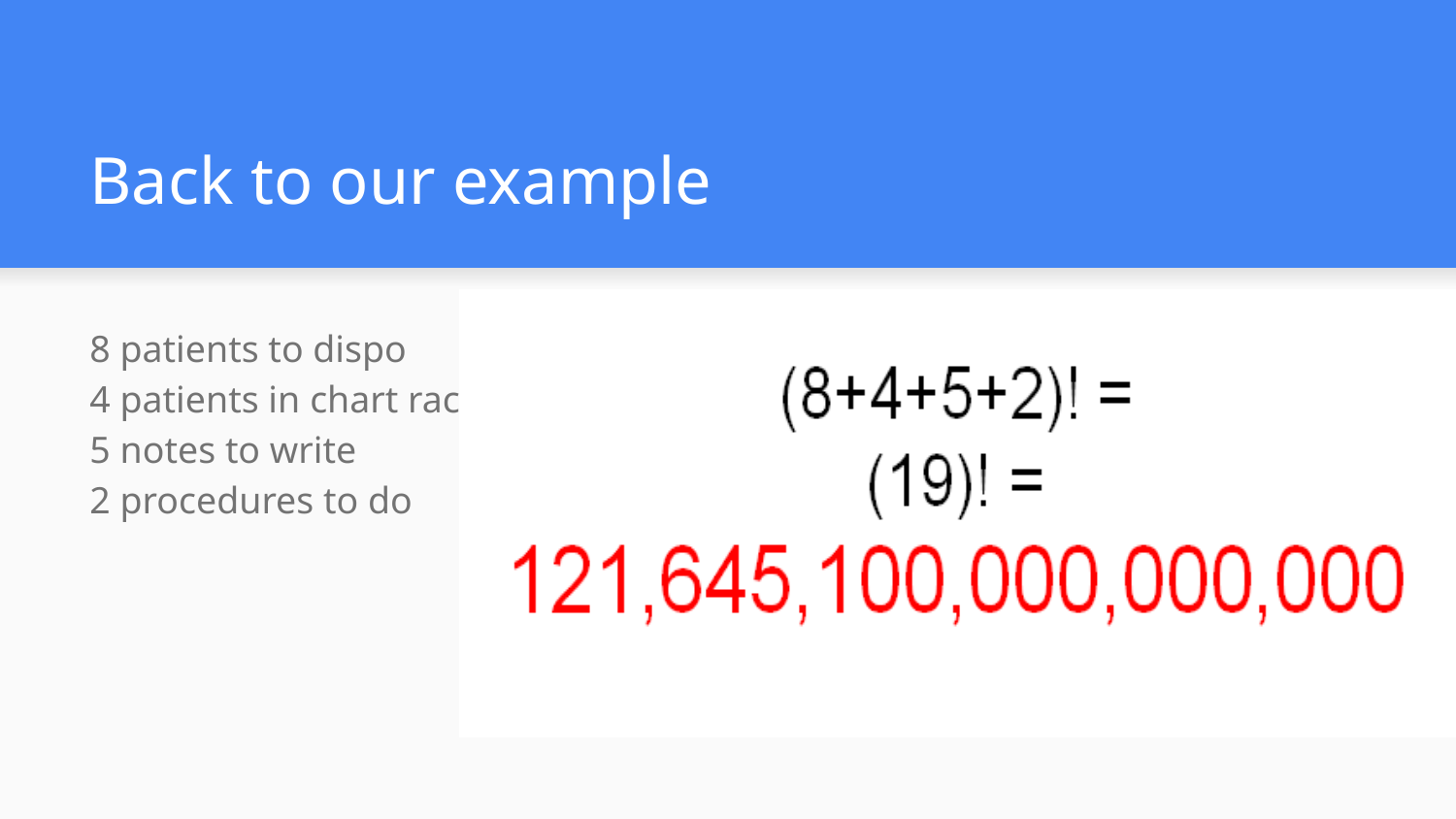

# Back to our example
8 patients to dispo
4 patients in chart rack
5 notes to write
2 procedures to do

## Slide 21
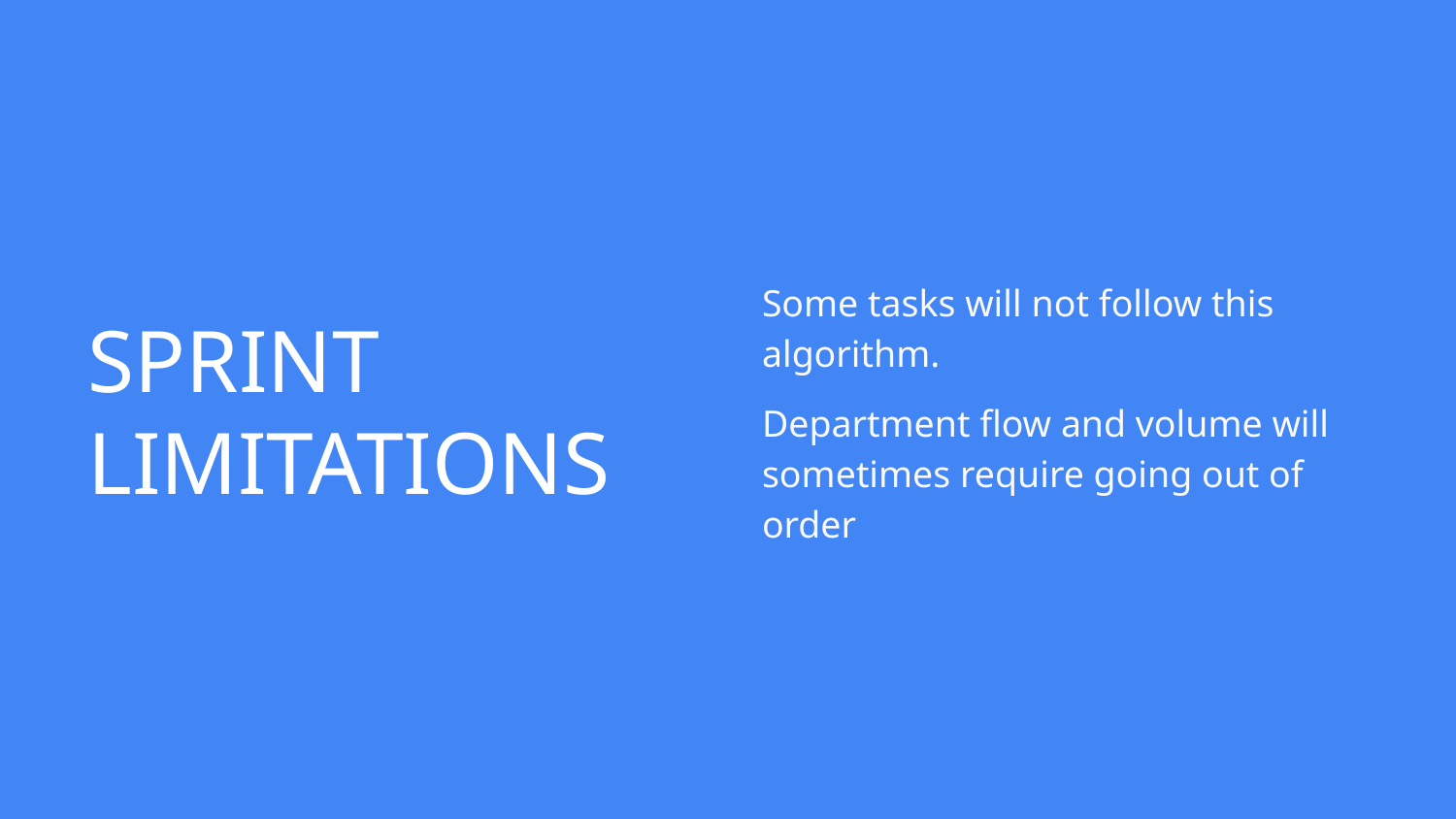

Some tasks will not follow this algorithm.
Department flow and volume will sometimes require going out of order
# SPRINT LIMITATIONS

## Slide 22
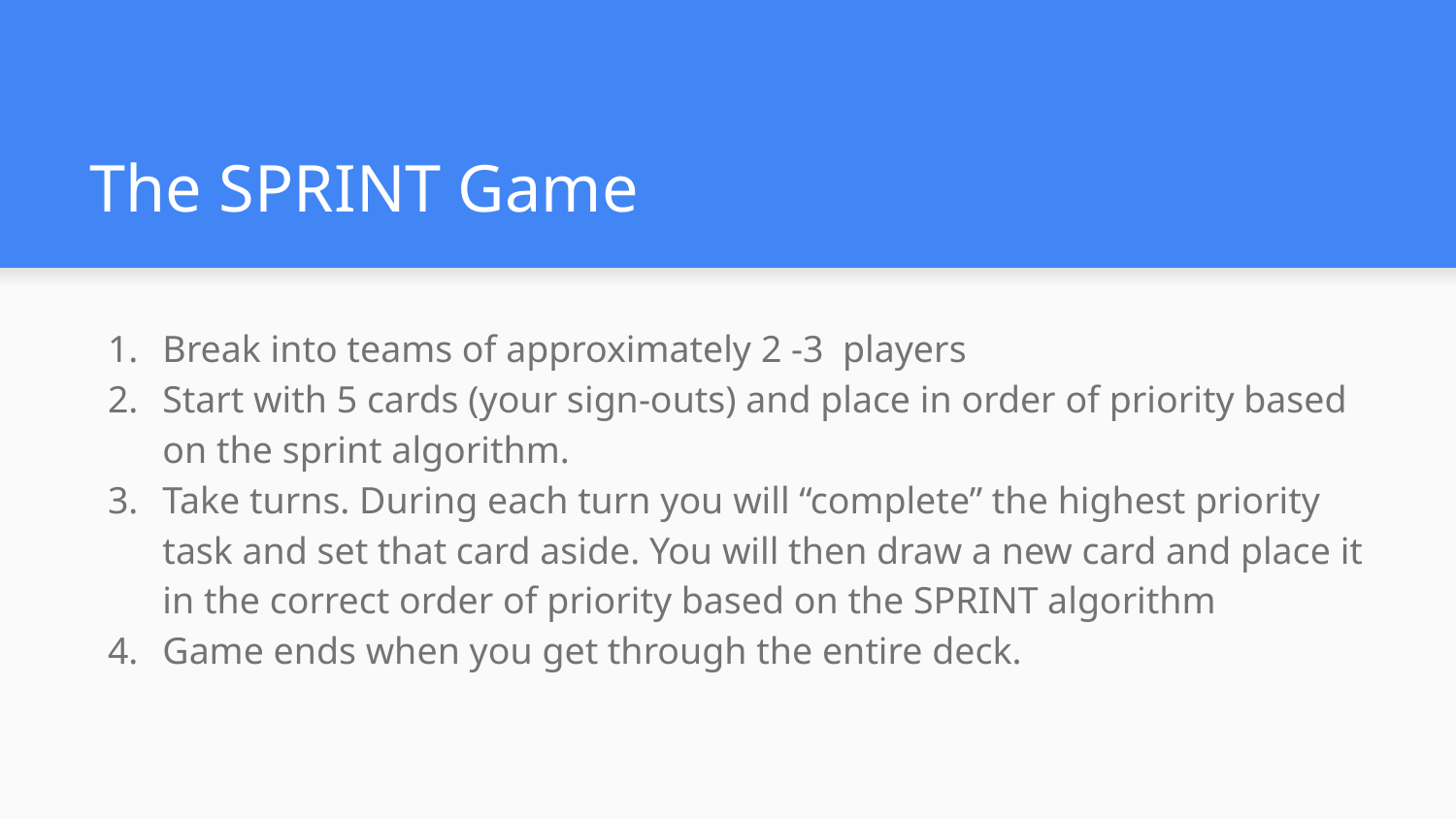

# The SPRINT Game
Break into teams of approximately 2 -3 players
Start with 5 cards (your sign-outs) and place in order of priority based on the sprint algorithm.
Take turns. During each turn you will “complete” the highest priority task and set that card aside. You will then draw a new card and place it in the correct order of priority based on the SPRINT algorithm
Game ends when you get through the entire deck.

## Slide 23
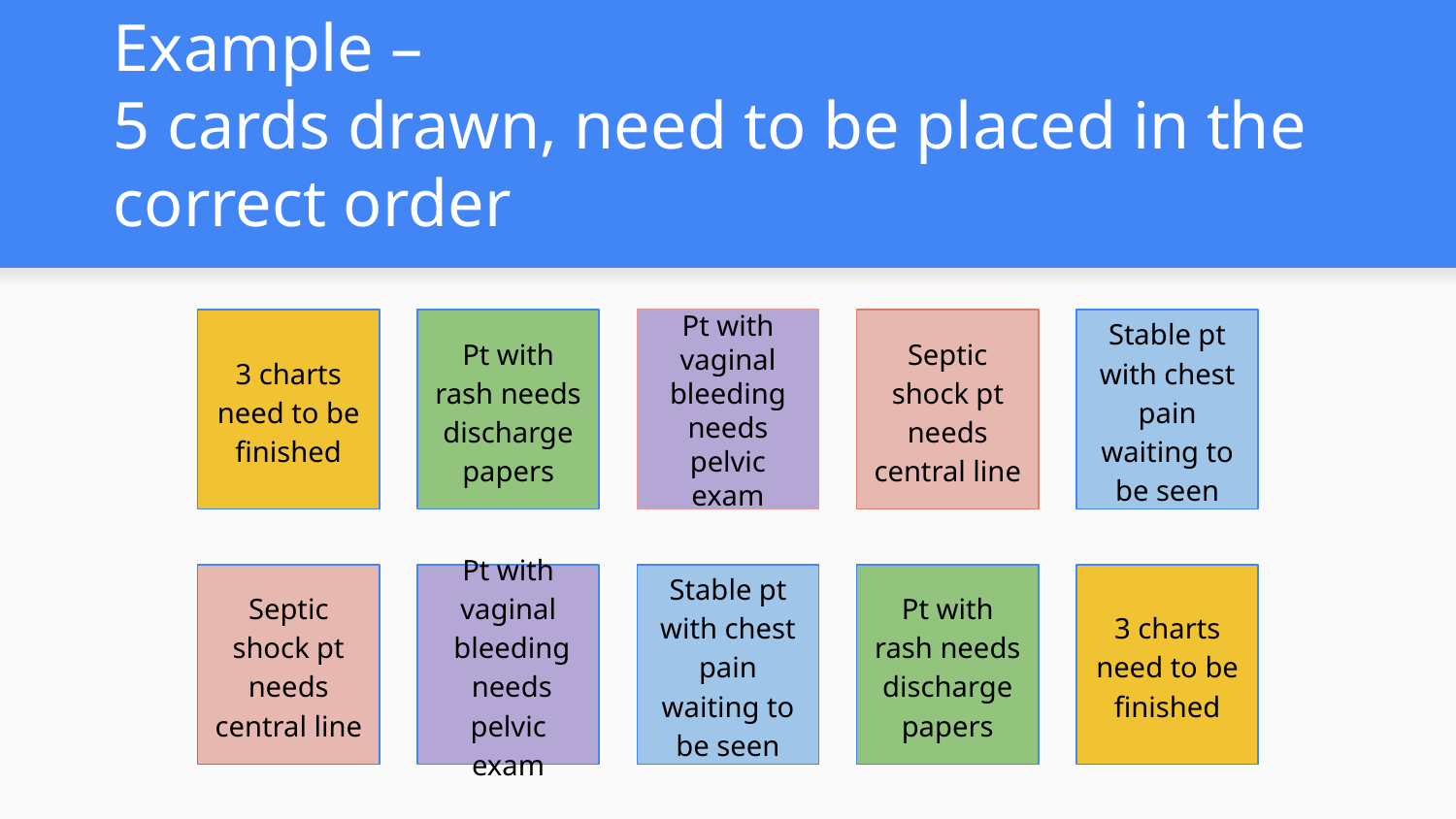

# Example – 5 cards drawn, need to be placed in the correct order
3 charts need to be finished
Pt with rash needs discharge papers
Pt with vaginal bleeding needs pelvic exam
Septic shock pt needs central line
Stable pt with chest pain waiting to be seen
Septic shock pt needs central line
Pt with vaginal
 bleeding
 needs pelvic exam
Stable pt with chest pain waiting to be seen
Pt with rash needs discharge papers
3 charts need to be finished

## Slide 24
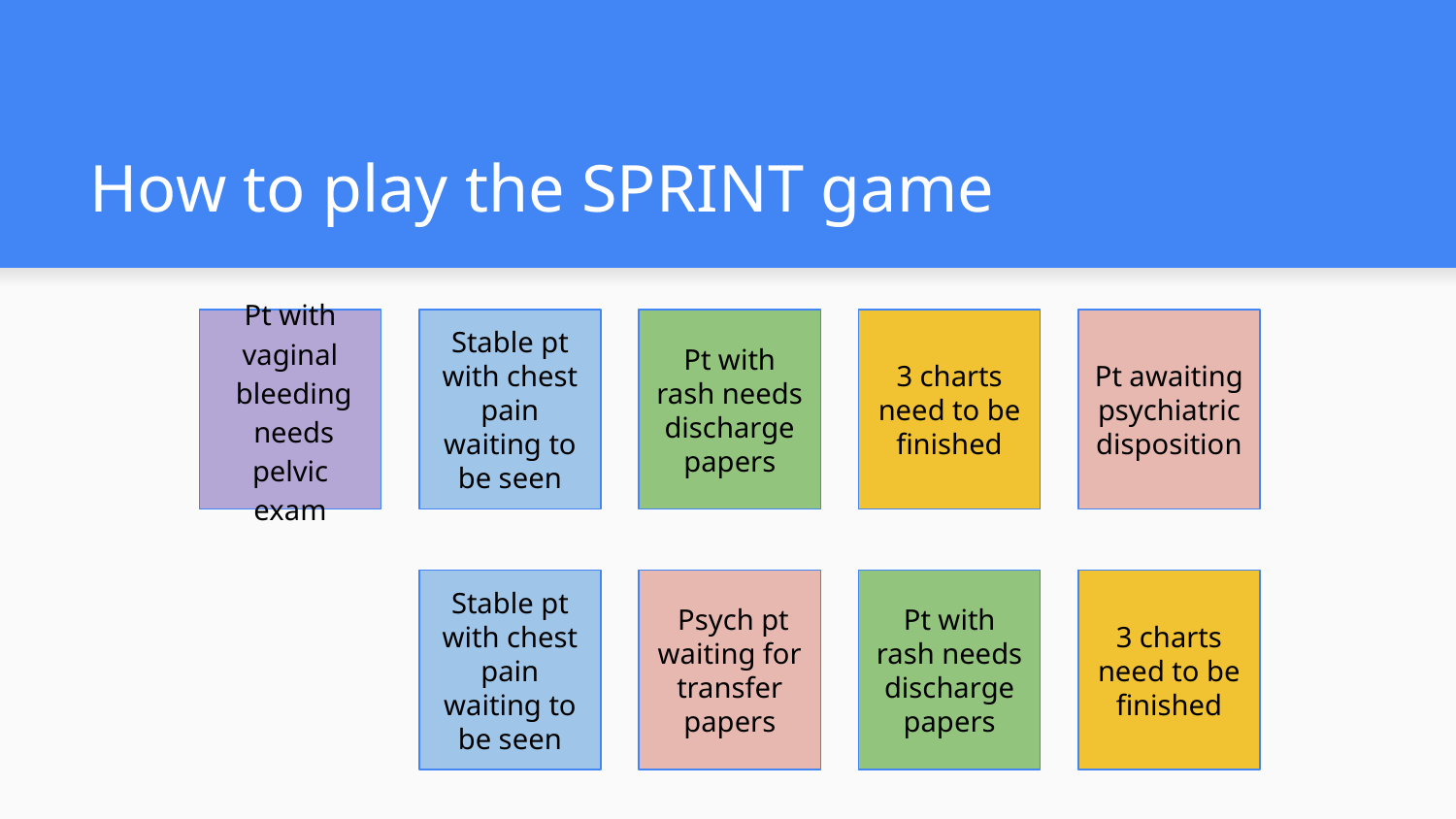

# How to play the SPRINT game
Pt with vaginal
 bleeding
 needs pelvic exam
Stable pt with chest pain waiting to be seen
Pt with rash needs discharge papers
3 charts need to be finished
Pt awaiting psychiatric disposition
Vaginal bleed needs pelvic exam
Stable pt with chest pain waiting to be seen
 Psych pt waiting for transfer papers
Pt with rash needs discharge papers
3 charts need to be finished

## Slide 25
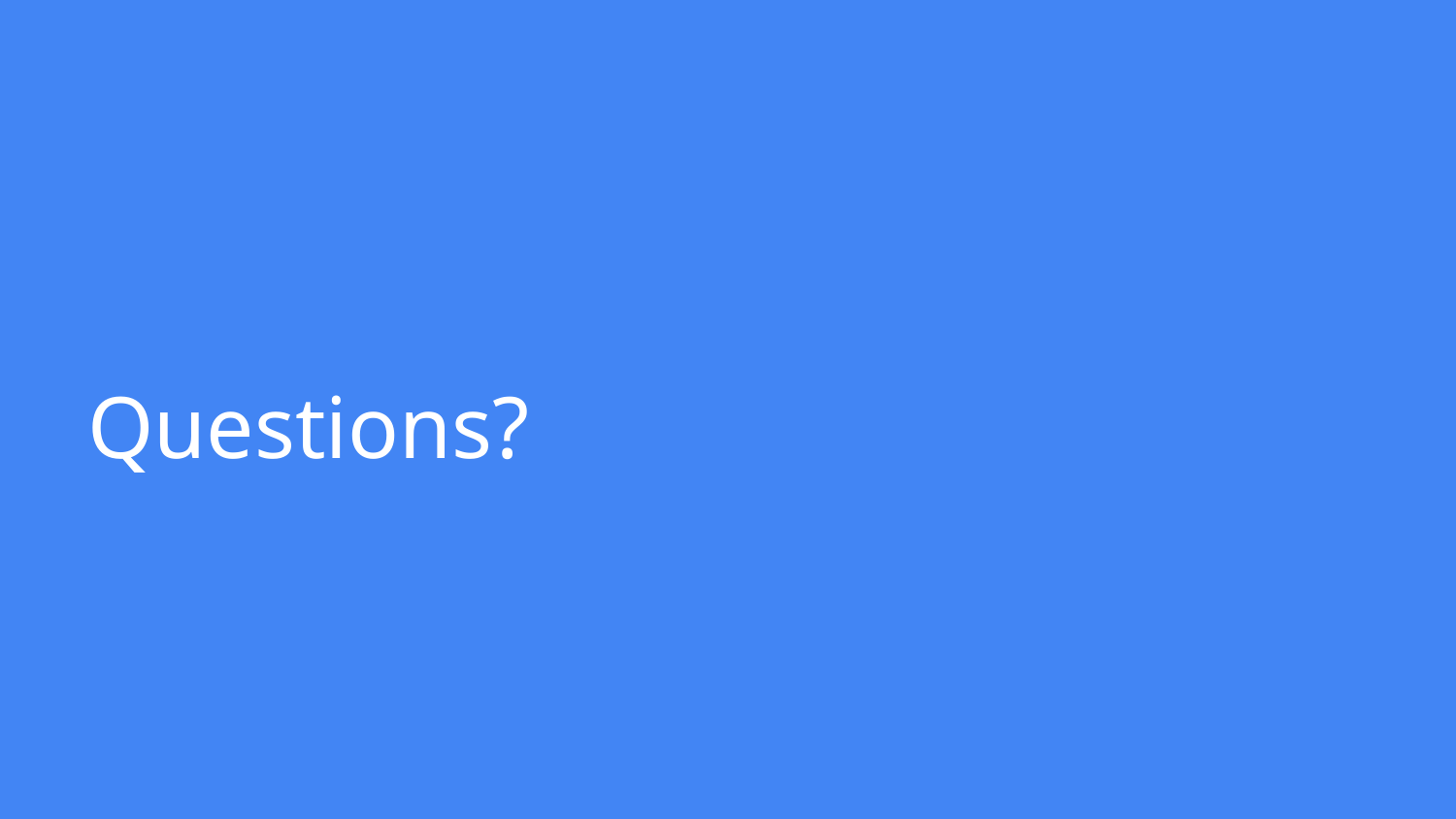

Efficiency Master
Workshop
Post-Test
# Questions?
