# Supplementary material for: SPRINT Through Tasks: A Novel Curriculum for Improving Resident Task Management in the Emergency Department
Source: MedEdPORTAL. 2020 Aug 25;16:10956. doi: 10.15766/mep_2374-8265.10956 (PMC7449580; doi:10.15766/mep_2374-8265.10956)
Supplement: Supplementary file 1 — Task Management in the ED.pptxSPRINT Video.mp4SPRINT Card Game.pptxSPRINT Badge Card.pdfSPRINT Preworkshop Survey.docxSPRINT Postworkshop Survey.docx [file mep_2374-8265.10956-s001.zip › C. SPRINT Card Game.pptx]

## Slide 1
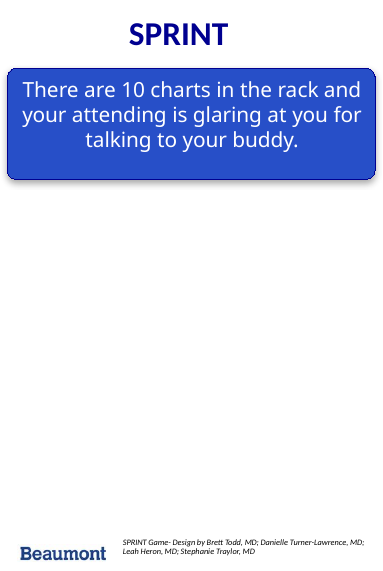

SPRINT
There are 10 charts in the rack and your attending is glaring at you for talking to your buddy.
SPRINT Game- Design by Brett Todd, MD; Danielle Turner-Lawrence, MD; Leah Heron, MD; Stephanie Traylor, MD

## Slide 2
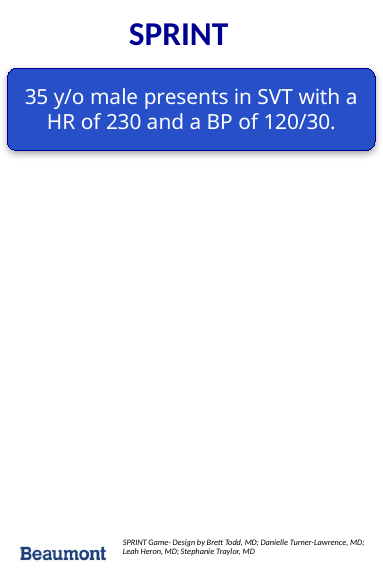

SPRINT
35 y/o male presents in SVT with a HR of 230 and a BP of 120/30.
SPRINT Game- Design by Brett Todd, MD; Danielle Turner-Lawrence, MD; Leah Heron, MD; Stephanie Traylor, MD

## Slide 3
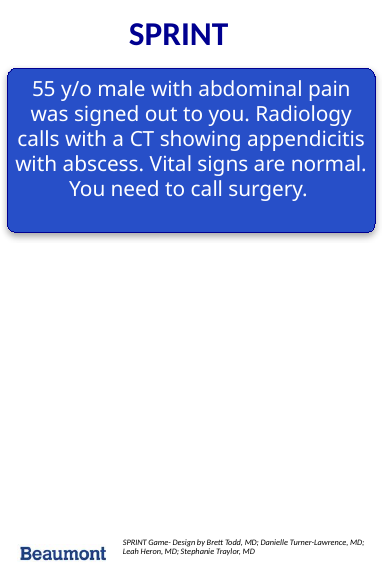

SPRINT
55 y/o male with abdominal pain was signed out to you. Radiology calls with a CT showing appendicitis with abscess. Vital signs are normal. You need to call surgery.
SPRINT Game- Design by Brett Todd, MD; Danielle Turner-Lawrence, MD; Leah Heron, MD; Stephanie Traylor, MD

## Slide 4
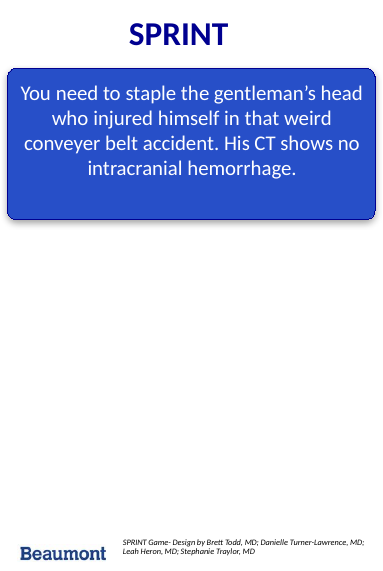

SPRINT
You need to staple the gentleman’s head who injured himself in that weird conveyer belt accident. His CT shows no intracranial hemorrhage.
SPRINT Game- Design by Brett Todd, MD; Danielle Turner-Lawrence, MD; Leah Heron, MD; Stephanie Traylor, MD

## Slide 5
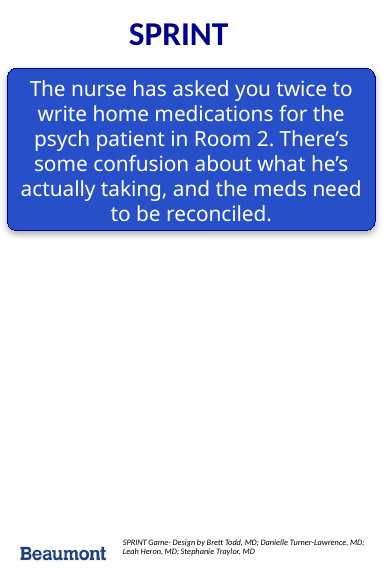

SPRINT
The nurse has asked you twice to write home medications for the psych patient in Room 2. There’s some confusion about what he’s actually taking, and the meds need to be reconciled.
SPRINT Game- Design by Brett Todd, MD; Danielle Turner-Lawrence, MD; Leah Heron, MD; Stephanie Traylor, MD

## Slide 6
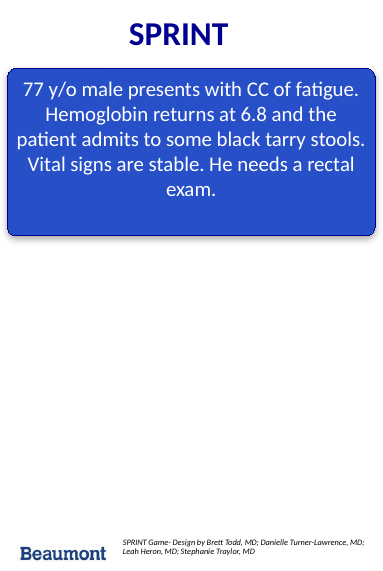

SPRINT
77 y/o male presents with CC of fatigue. Hemoglobin returns at 6.8 and the patient admits to some black tarry stools. Vital signs are stable. He needs a rectal exam.
SPRINT Game- Design by Brett Todd, MD; Danielle Turner-Lawrence, MD; Leah Heron, MD; Stephanie Traylor, MD

## Slide 7
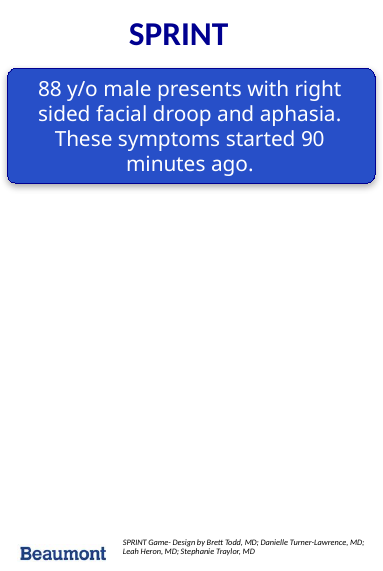

SPRINT
88 y/o male presents with right sided facial droop and aphasia. These symptoms started 90 minutes ago.
SPRINT Game- Design by Brett Todd, MD; Danielle Turner-Lawrence, MD; Leah Heron, MD; Stephanie Traylor, MD

## Slide 8
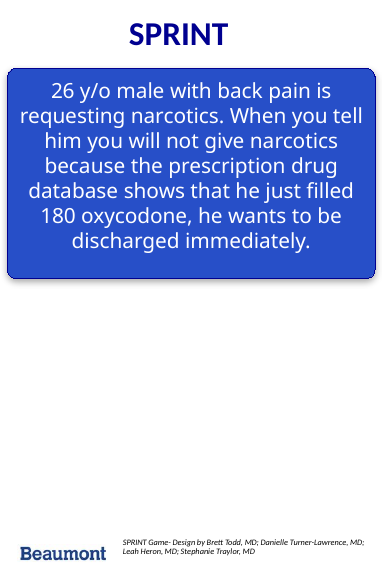

SPRINT
26 y/o male with back pain is requesting narcotics. When you tell him you will not give narcotics because the prescription drug database shows that he just filled 180 oxycodone, he wants to be discharged immediately.
SPRINT Game- Design by Brett Todd, MD; Danielle Turner-Lawrence, MD; Leah Heron, MD; Stephanie Traylor, MD

## Slide 9
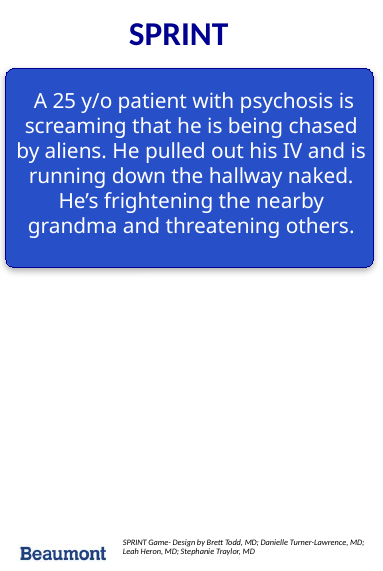

SPRINT
 A 25 y/o patient with psychosis is screaming that he is being chased by aliens. He pulled out his IV and is running down the hallway naked. He’s frightening the nearby grandma and threatening others.
SPRINT Game- Design by Brett Todd, MD; Danielle Turner-Lawrence, MD; Leah Heron, MD; Stephanie Traylor, MD

## Slide 10
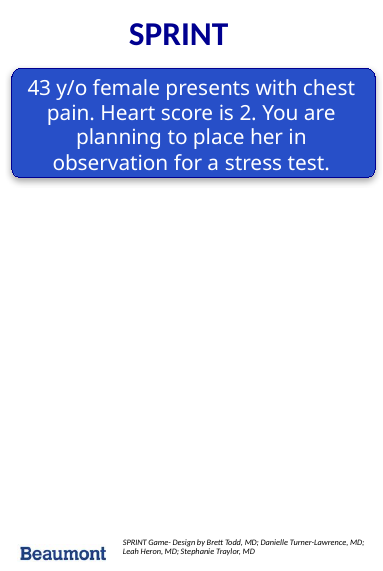

SPRINT
43 y/o female presents with chest pain. Heart score is 2. You are planning to place her in observation for a stress test.
SPRINT Game- Design by Brett Todd, MD; Danielle Turner-Lawrence, MD; Leah Heron, MD; Stephanie Traylor, MD

## Slide 11
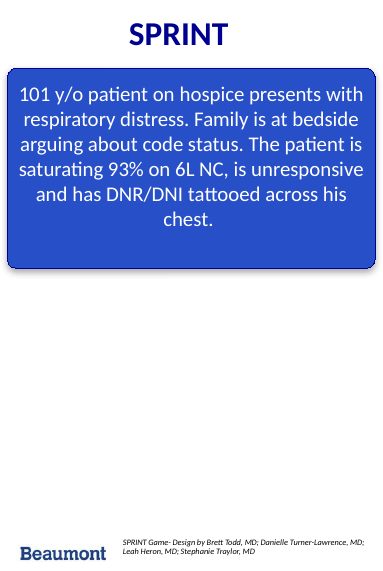

SPRINT
101 y/o patient on hospice presents with respiratory distress. Family is at bedside arguing about code status. The patient is saturating 93% on 6L NC, is unresponsive and has DNR/DNI tattooed across his chest.
SPRINT Game- Design by Brett Todd, MD; Danielle Turner-Lawrence, MD; Leah Heron, MD; Stephanie Traylor, MD

## Slide 12
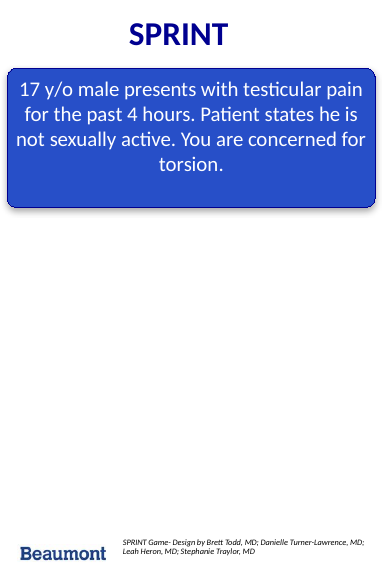

SPRINT
17 y/o male presents with testicular pain for the past 4 hours. Patient states he is not sexually active. You are concerned for torsion.
SPRINT Game- Design by Brett Todd, MD; Danielle Turner-Lawrence, MD; Leah Heron, MD; Stephanie Traylor, MD

## Slide 13
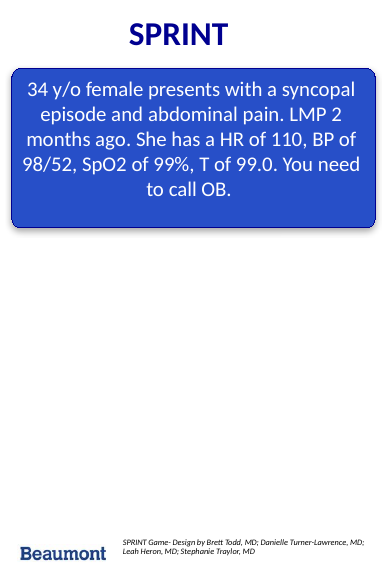

SPRINT
34 y/o female presents with a syncopal episode and abdominal pain. LMP 2 months ago. She has a HR of 110, BP of 98/52, SpO2 of 99%, T of 99.0. You need to call OB.
SPRINT Game- Design by Brett Todd, MD; Danielle Turner-Lawrence, MD; Leah Heron, MD; Stephanie Traylor, MD

## Slide 14
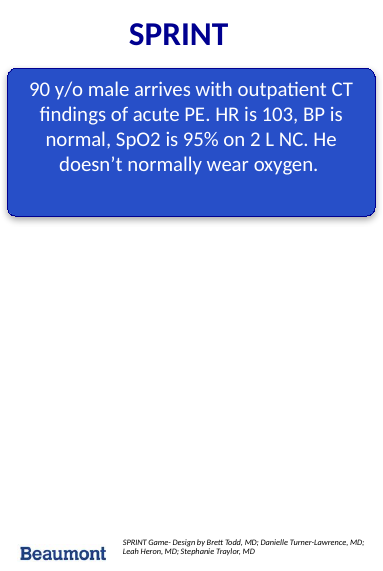

SPRINT
90 y/o male arrives with outpatient CT findings of acute PE. HR is 103, BP is normal, SpO2 is 95% on 2 L NC. He doesn’t normally wear oxygen.
SPRINT Game- Design by Brett Todd, MD; Danielle Turner-Lawrence, MD; Leah Heron, MD; Stephanie Traylor, MD

## Slide 15
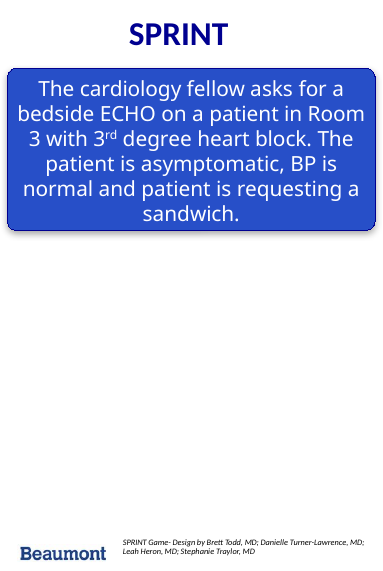

SPRINT
The cardiology fellow asks for a bedside ECHO on a patient in Room 3 with 3rd degree heart block. The patient is asymptomatic, BP is normal and patient is requesting a sandwich.
SPRINT Game- Design by Brett Todd, MD; Danielle Turner-Lawrence, MD; Leah Heron, MD; Stephanie Traylor, MD

## Slide 16
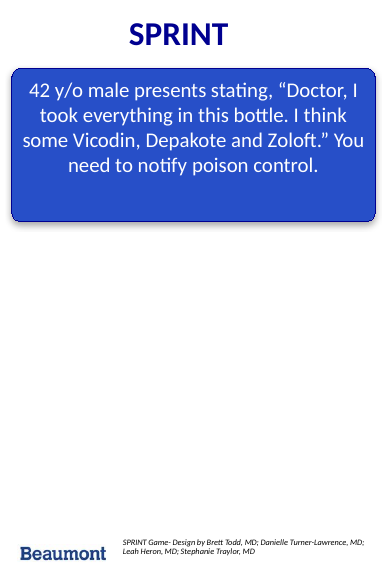

SPRINT
42 y/o male presents stating, “Doctor, I took everything in this bottle. I think some Vicodin, Depakote and Zoloft.” You need to notify poison control.
SPRINT Game- Design by Brett Todd, MD; Danielle Turner-Lawrence, MD; Leah Heron, MD; Stephanie Traylor, MD

## Slide 17
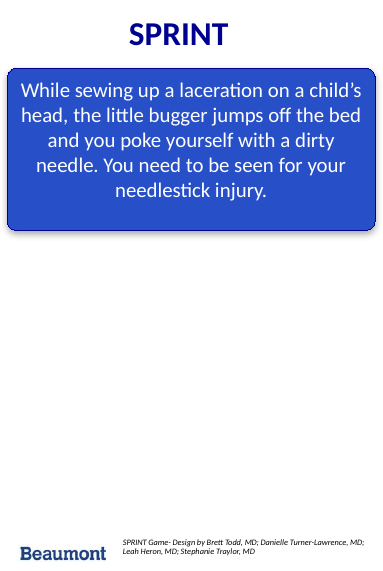

SPRINT
While sewing up a laceration on a child’s head, the little bugger jumps off the bed and you poke yourself with a dirty needle. You need to be seen for your needlestick injury.
SPRINT Game- Design by Brett Todd, MD; Danielle Turner-Lawrence, MD; Leah Heron, MD; Stephanie Traylor, MD

## Slide 18
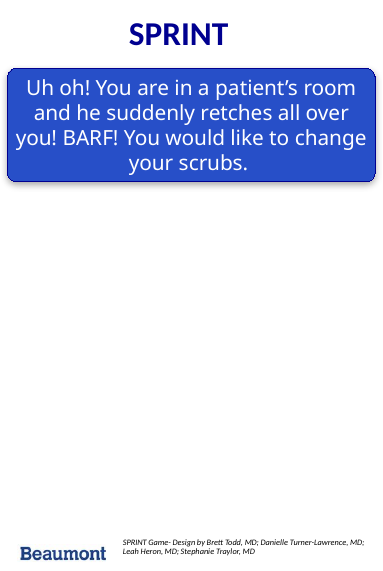

SPRINT
Uh oh! You are in a patient’s room and he suddenly retches all over you! BARF! You would like to change your scrubs.
SPRINT Game- Design by Brett Todd, MD; Danielle Turner-Lawrence, MD; Leah Heron, MD; Stephanie Traylor, MD

## Slide 19
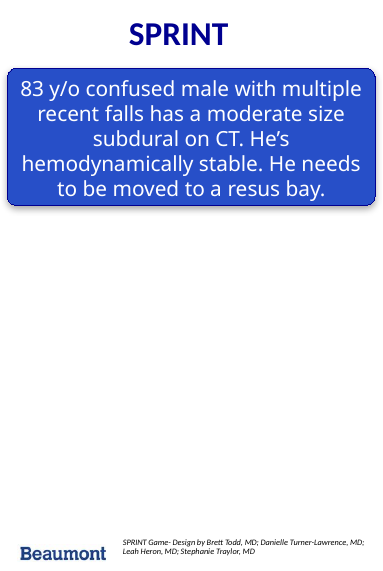

SPRINT
83 y/o confused male with multiple recent falls has a moderate size subdural on CT. He’s hemodynamically stable. He needs to be moved to a resus bay.
SPRINT Game- Design by Brett Todd, MD; Danielle Turner-Lawrence, MD; Leah Heron, MD; Stephanie Traylor, MD

## Slide 20
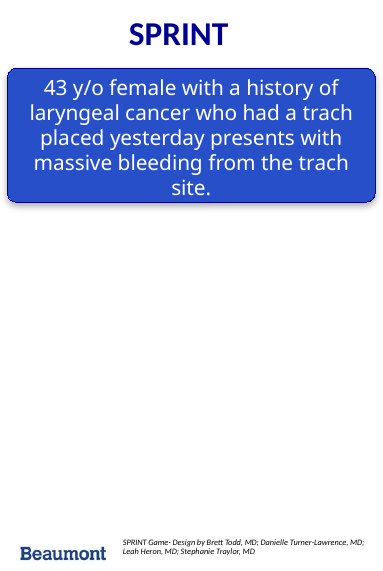

SPRINT
43 y/o female with a history of laryngeal cancer who had a trach placed yesterday presents with massive bleeding from the trach site.
SPRINT Game- Design by Brett Todd, MD; Danielle Turner-Lawrence, MD; Leah Heron, MD; Stephanie Traylor, MD

## Slide 21
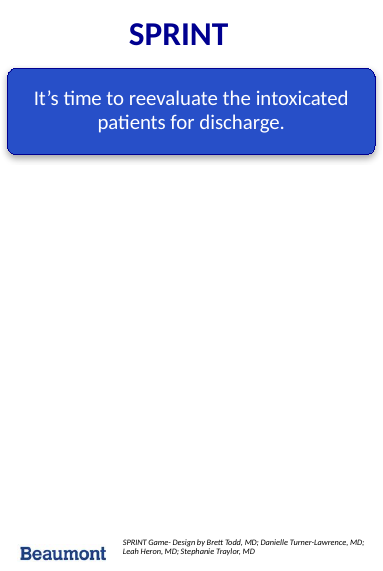

SPRINT
It’s time to reevaluate the intoxicated patients for discharge.
SPRINT Game- Design by Brett Todd, MD; Danielle Turner-Lawrence, MD; Leah Heron, MD; Stephanie Traylor, MD

## Slide 22
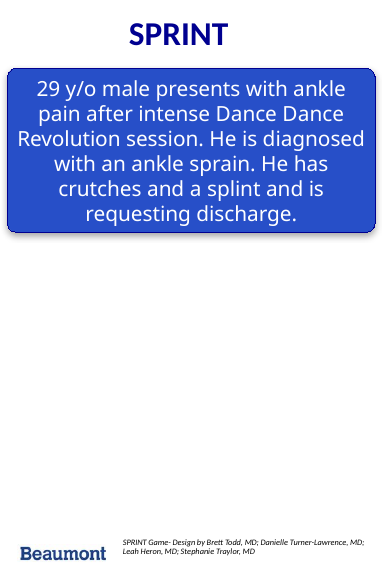

SPRINT
29 y/o male presents with ankle pain after intense Dance Dance Revolution session. He is diagnosed with an ankle sprain. He has crutches and a splint and is requesting discharge.
SPRINT Game- Design by Brett Todd, MD; Danielle Turner-Lawrence, MD; Leah Heron, MD; Stephanie Traylor, MD

## Slide 23
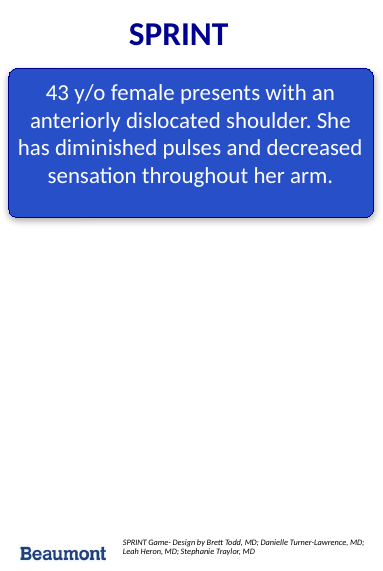

SPRINT
43 y/o female presents with an anteriorly dislocated shoulder. She has diminished pulses and decreased sensation throughout her arm.
SPRINT Game- Design by Brett Todd, MD; Danielle Turner-Lawrence, MD; Leah Heron, MD; Stephanie Traylor, MD

## Slide 24
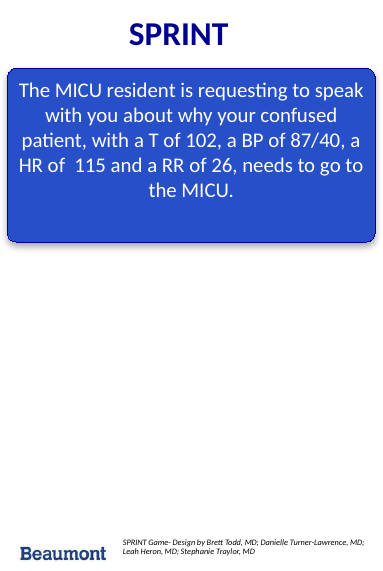

SPRINT
The MICU resident is requesting to speak with you about why your confused patient, with a T of 102, a BP of 87/40, a HR of 115 and a RR of 26, needs to go to the MICU.
SPRINT Game- Design by Brett Todd, MD; Danielle Turner-Lawrence, MD; Leah Heron, MD; Stephanie Traylor, MD

## Slide 25
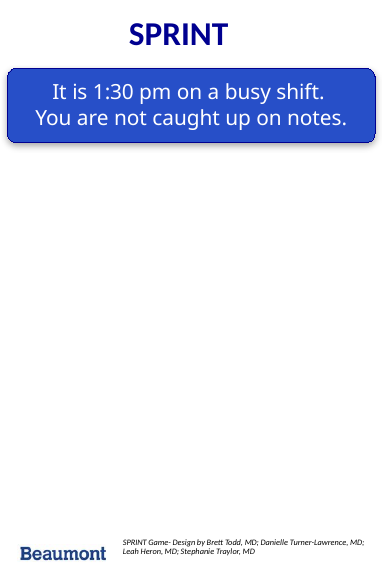

SPRINT
It is 1:30 pm on a busy shift.
You are not caught up on notes.
SPRINT Game- Design by Brett Todd, MD; Danielle Turner-Lawrence, MD; Leah Heron, MD; Stephanie Traylor, MD

## Slide 26
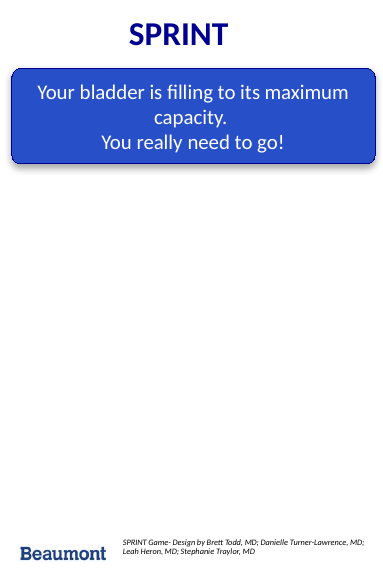

SPRINT
Your bladder is filling to its maximum capacity.
You really need to go!
SPRINT Game- Design by Brett Todd, MD; Danielle Turner-Lawrence, MD; Leah Heron, MD; Stephanie Traylor, MD

## Slide 27
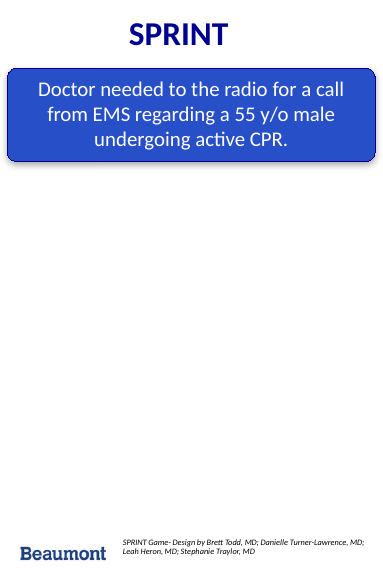

SPRINT
Doctor needed to the radio for a call from EMS regarding a 55 y/o male undergoing active CPR.
SPRINT Game- Design by Brett Todd, MD; Danielle Turner-Lawrence, MD; Leah Heron, MD; Stephanie Traylor, MD

## Slide 28
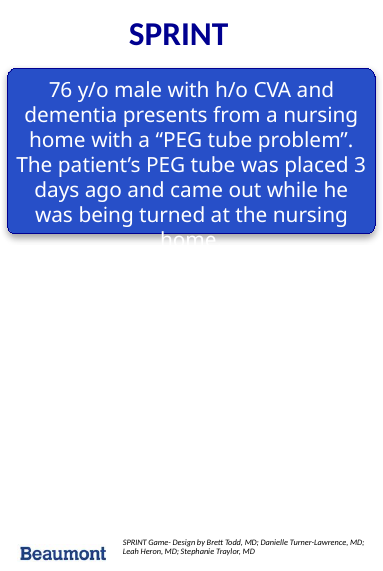

SPRINT
76 y/o male with h/o CVA and dementia presents from a nursing home with a “PEG tube problem”. The patient’s PEG tube was placed 3 days ago and came out while he was being turned at the nursing home.
SPRINT Game- Design by Brett Todd, MD; Danielle Turner-Lawrence, MD; Leah Heron, MD; Stephanie Traylor, MD

## Slide 29
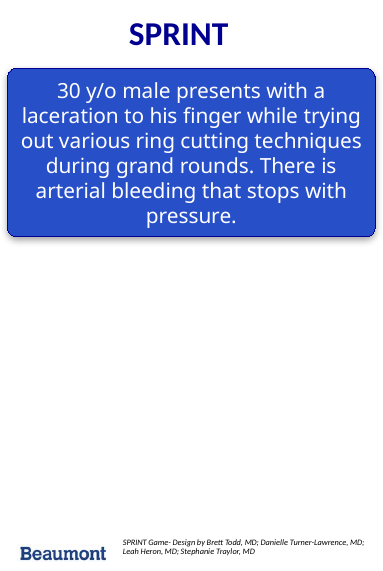

SPRINT
30 y/o male presents with a laceration to his finger while trying out various ring cutting techniques during grand rounds. There is arterial bleeding that stops with pressure.
SPRINT Game- Design by Brett Todd, MD; Danielle Turner-Lawrence, MD; Leah Heron, MD; Stephanie Traylor, MD

## Slide 30
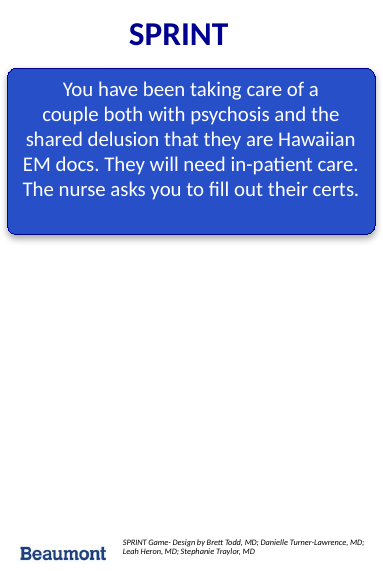

SPRINT
You have been taking care of a couple both with psychosis and the shared delusion that they are Hawaiian EM docs. They will need in-patient care. The nurse asks you to fill out their certs.
SPRINT Game- Design by Brett Todd, MD; Danielle Turner-Lawrence, MD; Leah Heron, MD; Stephanie Traylor, MD

## Slide 31
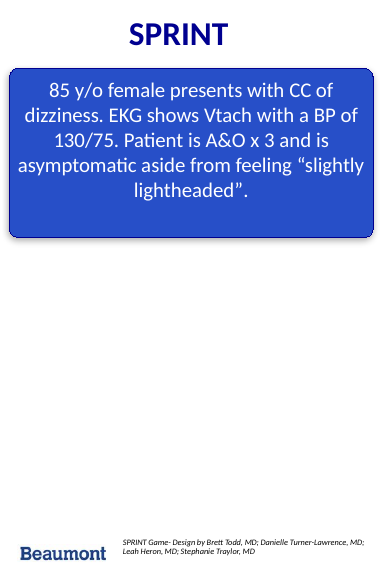

SPRINT
85 y/o female presents with CC of dizziness. EKG shows Vtach with a BP of 130/75. Patient is A&O x 3 and is asymptomatic aside from feeling “slightly lightheaded”.
SPRINT Game- Design by Brett Todd, MD; Danielle Turner-Lawrence, MD; Leah Heron, MD; Stephanie Traylor, MD

## Slide 32
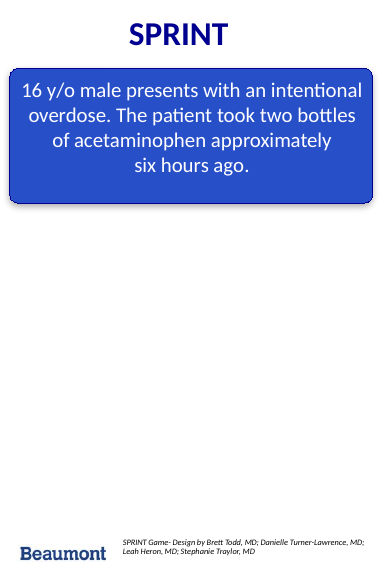

SPRINT
16 y/o male presents with an intentional overdose. The patient took two bottles of acetaminophen approximately
six hours ago.
SPRINT Game- Design by Brett Todd, MD; Danielle Turner-Lawrence, MD; Leah Heron, MD; Stephanie Traylor, MD

## Slide 33
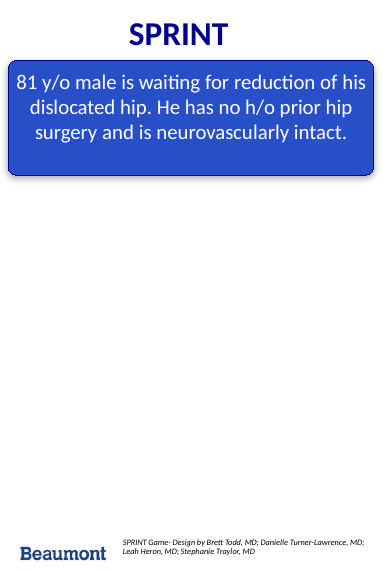

SPRINT
81 y/o male is waiting for reduction of his dislocated hip. He has no h/o prior hip surgery and is neurovascularly intact.
SPRINT Game- Design by Brett Todd, MD; Danielle Turner-Lawrence, MD; Leah Heron, MD; Stephanie Traylor, MD

## Slide 34
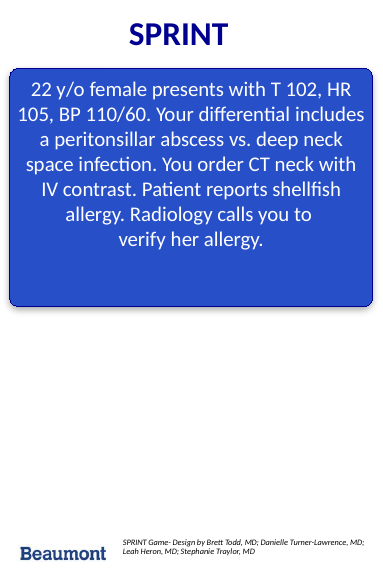

SPRINT
22 y/o female presents with T 102, HR 105, BP 110/60. Your differential includes a peritonsillar abscess vs. deep neck space infection. You order CT neck with IV contrast. Patient reports shellfish allergy. Radiology calls you to
verify her allergy.
SPRINT Game- Design by Brett Todd, MD; Danielle Turner-Lawrence, MD; Leah Heron, MD; Stephanie Traylor, MD

## Slide 35
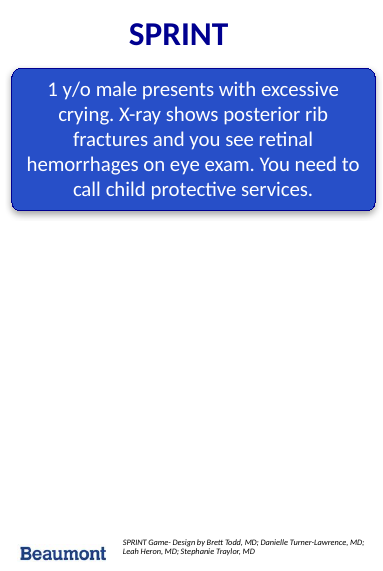

SPRINT
1 y/o male presents with excessive crying. X-ray shows posterior rib fractures and you see retinal hemorrhages on eye exam. You need to call child protective services.
SPRINT Game- Design by Brett Todd, MD; Danielle Turner-Lawrence, MD; Leah Heron, MD; Stephanie Traylor, MD

## Slide 36
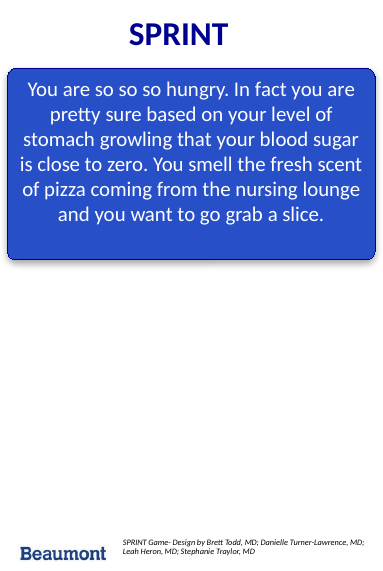

SPRINT
You are so so so hungry. In fact you are pretty sure based on your level of stomach growling that your blood sugar is close to zero. You smell the fresh scent of pizza coming from the nursing lounge and you want to go grab a slice.
SPRINT Game- Design by Brett Todd, MD; Danielle Turner-Lawrence, MD; Leah Heron, MD; Stephanie Traylor, MD

## Slide 37
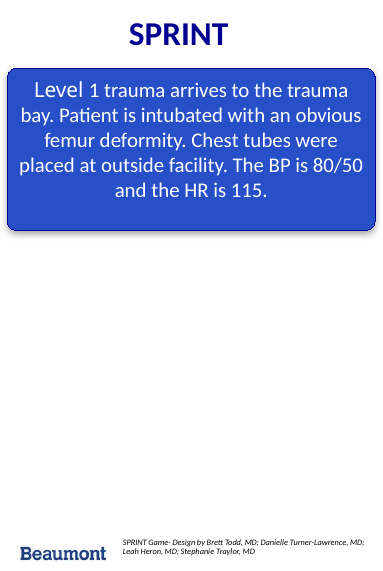

SPRINT
Level 1 trauma arrives to the trauma bay. Patient is intubated with an obvious femur deformity. Chest tubes were placed at outside facility. The BP is 80/50 and the HR is 115.
SPRINT Game- Design by Brett Todd, MD; Danielle Turner-Lawrence, MD; Leah Heron, MD; Stephanie Traylor, MD

## Slide 38
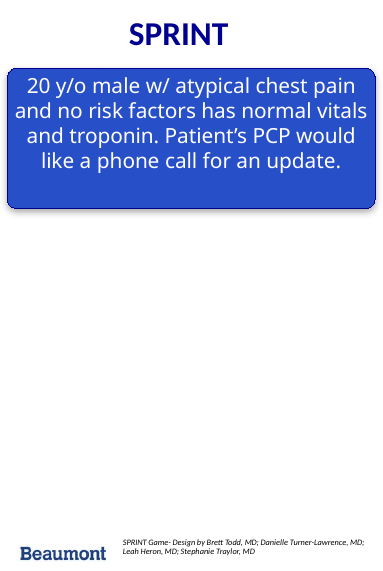

SPRINT
20 y/o male w/ atypical chest pain and no risk factors has normal vitals and troponin. Patient’s PCP would like a phone call for an update.
SPRINT Game- Design by Brett Todd, MD; Danielle Turner-Lawrence, MD; Leah Heron, MD; Stephanie Traylor, MD

## Slide 39
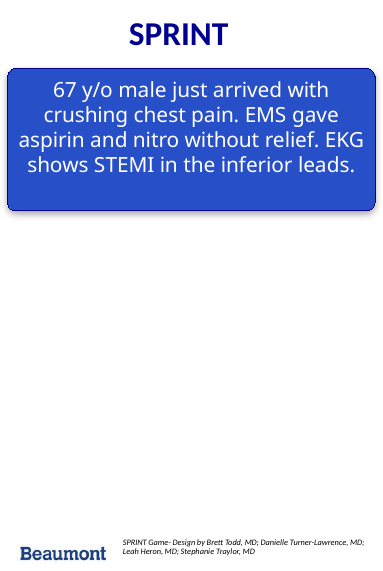

SPRINT
67 y/o male just arrived with crushing chest pain. EMS gave aspirin and nitro without relief. EKG shows STEMI in the inferior leads.
SPRINT Game- Design by Brett Todd, MD; Danielle Turner-Lawrence, MD; Leah Heron, MD; Stephanie Traylor, MD

## Slide 40
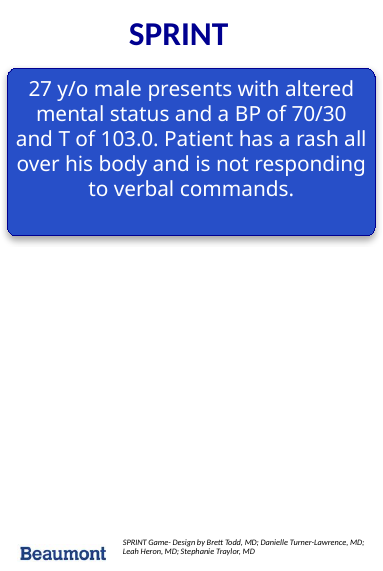

SPRINT
27 y/o male presents with altered mental status and a BP of 70/30 and T of 103.0. Patient has a rash all over his body and is not responding to verbal commands.
SPRINT Game- Design by Brett Todd, MD; Danielle Turner-Lawrence, MD; Leah Heron, MD; Stephanie Traylor, MD

## Slide 41
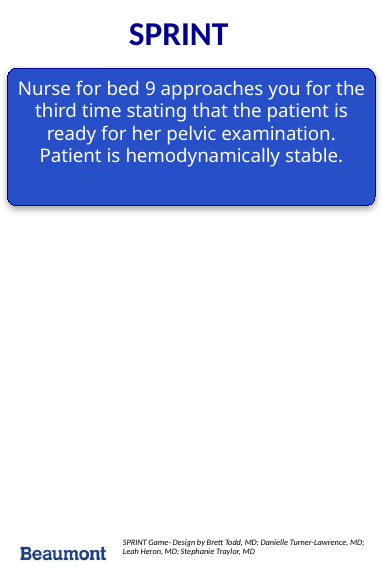

SPRINT
Nurse for bed 9 approaches you for the third time stating that the patient is ready for her pelvic examination. Patient is hemodynamically stable.
SPRINT Game- Design by Brett Todd, MD; Danielle Turner-Lawrence, MD; Leah Heron, MD; Stephanie Traylor, MD

## Slide 42
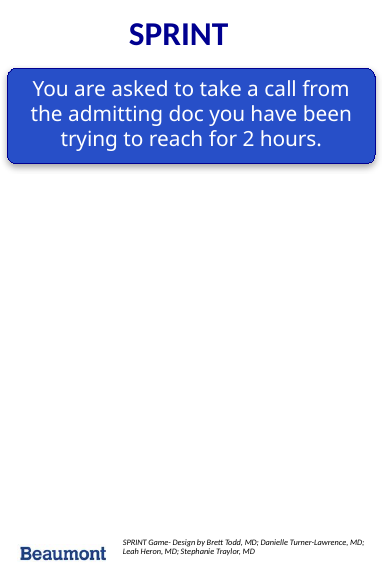

SPRINT
You are asked to take a call from the admitting doc you have been trying to reach for 2 hours.
SPRINT Game- Design by Brett Todd, MD; Danielle Turner-Lawrence, MD; Leah Heron, MD; Stephanie Traylor, MD

## Slide 43
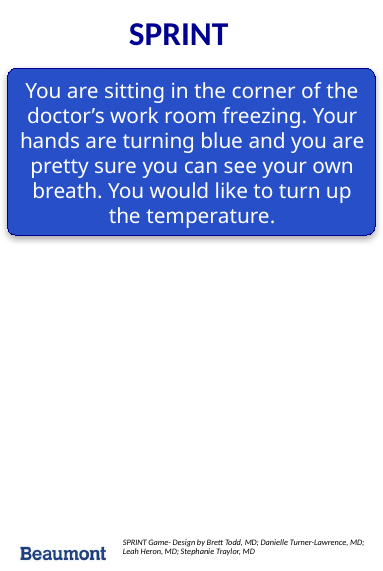

SPRINT
You are sitting in the corner of the doctor’s work room freezing. Your hands are turning blue and you are pretty sure you can see your own breath. You would like to turn up the temperature.
SPRINT Game- Design by Brett Todd, MD; Danielle Turner-Lawrence, MD; Leah Heron, MD; Stephanie Traylor, MD

## Slide 44
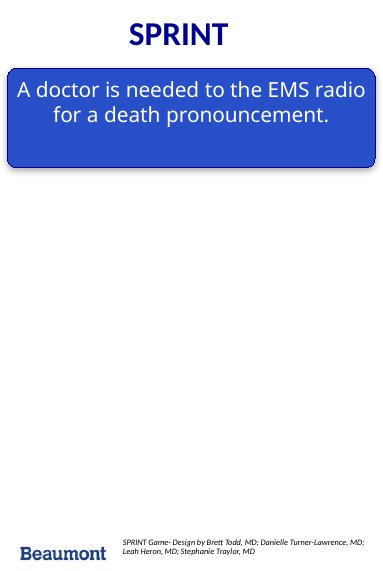

SPRINT
A doctor is needed to the EMS radio for a death pronouncement.
SPRINT Game- Design by Brett Todd, MD; Danielle Turner-Lawrence, MD; Leah Heron, MD; Stephanie Traylor, MD

## Slide 45
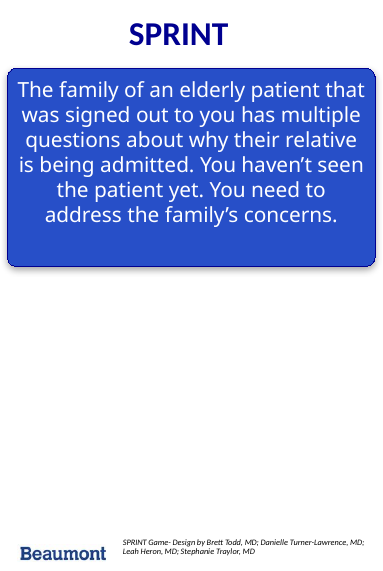

SPRINT
The family of an elderly patient that was signed out to you has multiple questions about why their relative is being admitted. You haven’t seen the patient yet. You need to address the family’s concerns.
SPRINT Game- Design by Brett Todd, MD; Danielle Turner-Lawrence, MD; Leah Heron, MD; Stephanie Traylor, MD

## Slide 46
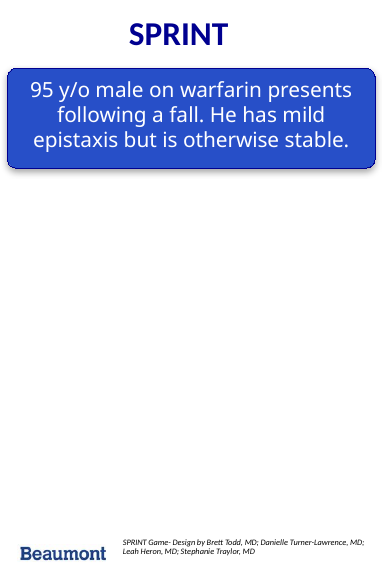

SPRINT
95 y/o male on warfarin presents following a fall. He has mild epistaxis but is otherwise stable.
SPRINT Game- Design by Brett Todd, MD; Danielle Turner-Lawrence, MD; Leah Heron, MD; Stephanie Traylor, MD

## Slide 47
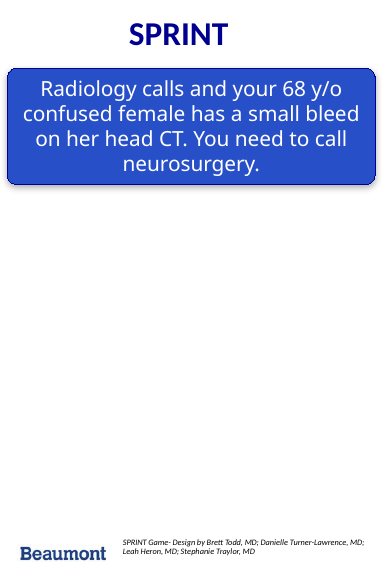

SPRINT
Radiology calls and your 68 y/o confused female has a small bleed on her head CT. You need to call neurosurgery.
SPRINT Game- Design by Brett Todd, MD; Danielle Turner-Lawrence, MD; Leah Heron, MD; Stephanie Traylor, MD

## Slide 48
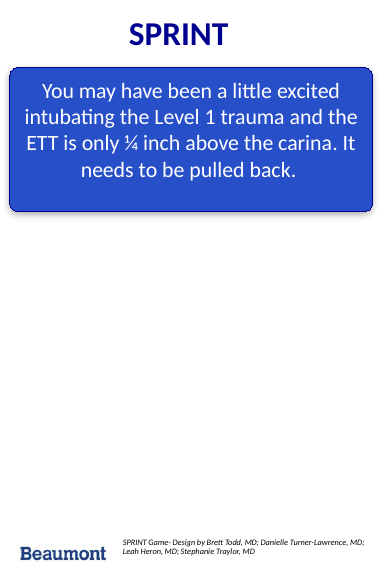

SPRINT
You may have been a little excited intubating the Level 1 trauma and the ETT is only ¼ inch above the carina. It needs to be pulled back.
SPRINT Game- Design by Brett Todd, MD; Danielle Turner-Lawrence, MD; Leah Heron, MD; Stephanie Traylor, MD

## Slide 49
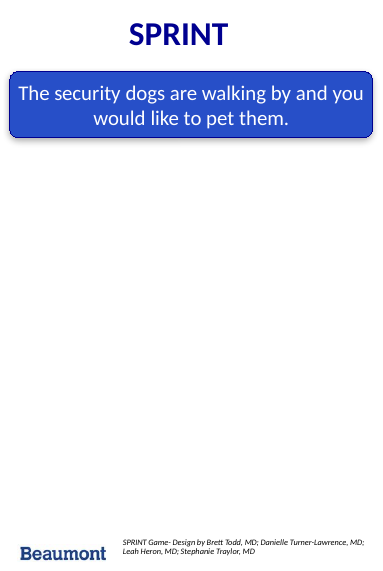

SPRINT
The security dogs are walking by and you would like to pet them.
SPRINT Game- Design by Brett Todd, MD; Danielle Turner-Lawrence, MD; Leah Heron, MD; Stephanie Traylor, MD
