# Supplementary material for: SPRINT Through Tasks: A Novel Curriculum for Improving Resident Task Management in the Emergency Department
Source: MedEdPORTAL. 2020 Aug 25;16:10956. doi: 10.15766/mep_2374-8265.10956 (PMC7449580; doi:10.15766/mep_2374-8265.10956)
Supplement: Supplementary file 1 — Task Management in the ED.pptxSPRINT Video.mp4SPRINT Card Game.pptxSPRINT Badge Card.pdfSPRINT Preworkshop Survey.docxSPRINT Postworkshop Survey.docx [file mep_2374-8265.10956-s001.zip › D. SPRINT Badge Card.pdf]

SPRINT

Stabilize
- Stabilize critical patients

Procedures
- Do non-emergent procedures

Rack
- See new patients

IN or out
- Reassess and dispostion

Type it up
- Chart completion

Beaumont
