# Supplementary material for: SPRINT Through Tasks: A Novel Curriculum for Improving Resident Task Management in the Emergency Department
Source: MedEdPORTAL. 2020 Aug 25;16:10956. doi: 10.15766/mep_2374-8265.10956 (PMC7449580; doi:10.15766/mep_2374-8265.10956)
Supplement: Supplementary file 1 — Task Management in the ED.pptxSPRINT Video.mp4SPRINT Card Game.pptxSPRINT Badge Card.pdfSPRINT Preworkshop Survey.docxSPRINT Postworkshop Survey.docx [file mep_2374-8265.10956-s001.zip › F. SPRINT Postworkshop Survey.docx]

Task Management in the ED

Postworkshop Assessment

This is a single-center, prospective cohort study. Please feel free to answer all questions. This survey is confidential and anonymous. It has no effect on your performance evaluations. This data may be used to assess the effectiveness of a residency training curriculum. Thank you very much for your participation.

1. I am satisfied with my ability to manage tasks efficiently in the Emergency Department.
   - Strongly Agree
   - Agree
   - Neutral
   - Disagree
   - Strongly disagree
2. I have difficulty prioritizing tasks.
   - Strongly Agree
   - Agree
   - Neutral
   - Disagree
   - Strongly disagree
3. I feel overwhelmed by the number of tasks I have on shift.
   - Strongly agree
   - Agree
   - Neutral
   - Disagree
   - Strongly disagree
4. I have a strategy for task prioritization.
   - Strongly Agree
   - Agree
   - Neutral
   - Disagree
   - Strongly disagree
5. I am usually able to leave my shift on time.
   - Strongly agree
   - Agree
   - Neutral
   - Disagree
   - Strongly disagree
6. I believe the SPRINT decision tool is effective in helping me with task management.
   - Strongly agree
   - Agree
   - Neutral
   - Disagree
   - Strongly disagree
7. I thought the decision overload workshop (or video) was engaging/enjoyable.
   - Strongly agree
   - Agree
   - Neutral
   - Disagree
   - Strongly disagree
8. I feel more confident about managing tasks in the ED after attending the workshop (or watching the video) on multitasking.
   - Strongly agree
   - Agree
   - Neutral
   - Disagree
   - Strongly disagree
9. I applied the SPRINT decision tool on shift.
   - Strongly agree
   - Agree
   - Neutral
   - Disagree
   - Strongly disagree
